# Skin cancer detection using non-invasive techniques

**DOI:** 10.1039/c8ra04164d

**Published:** 2018-08-06

**Authors:** Vigneswaran Narayanamurthy, P. Padmapriya, A. Noorasafrin, B. Pooja, K. Hema, Al'aina Yuhainis Firus Khan, K. Nithyakalyani, Fahmi Samsuri

**Affiliations:** InnoFuTech No: 42/12, 7th Street, Vallalar Nagar, Pattabiram Chennai Tamil Nadu 600072 India PEL13006@stdmail.ump.edu.my; Faculty of Electrical and Electronics Engineering, University Malaysia Pahang Pekan 26600 Malaysia; Department of Biomedical Engineering, Veltech Multitech Dr. RR & Dr. SR Engineering College Chennai 600 062 India; Department of Biomedical Science, Faculty of Allied Health Sciences, International Islamic University Malaysia 25200 Kuantan Pahang Malaysia

## Abstract

Skin cancer is the most common form of cancer and is globally rising. Historically, the diagnosis of skin cancers has depended on various conventional techniques which are of an invasive manner. A variety of commercial diagnostic tools and auxiliary techniques are available to detect skin cancer. This article explains in detail the principles and approaches involved for non-invasive skin cancer diagnostic methods such as photography, dermoscopy, sonography, confocal microscopy, Raman spectroscopy, fluorescence spectroscopy, terahertz spectroscopy, optical coherence tomography, the multispectral imaging technique, thermography, electrical bio-impedance, tape stripping and computer-aided analysis. The characteristics of an ideal screening test are outlined, and the authors pose several points for clinicians and scientists to consider in the evaluation of current and future studies of skin cancer detection and diagnosis. This comprehensive review critically analyses the literature associated with the field and summarises the recent updates along with their merits and demerits.

## Abstract 2

Recent advances in non-invasive techniques for skin cancer diagnosis.

## Graphical abstract

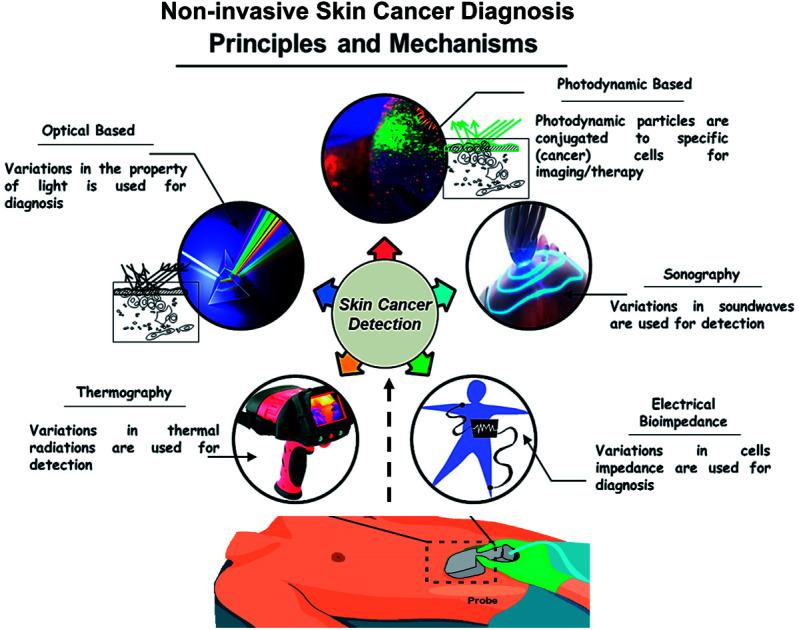

## Introduction

Skin cancer is a common type of cancer that emerges from the skin. It is extended due to the development of abnormal growth of cells. These cells can invade other parts of the body. More than 90% of cases occur due to the exposure of UV radiation. The UV radiation wavelength ranges from 100 nm to 400 nm. Cancer occurs due to unrepaired DNA damage to skin cells which is caused mostly due to UV radiation. In melanoma, cancer cells arise from moles on the skin which causes inflammation around the epidermal layer which in turn increases the temperature around it.^1^

**Fig. 1:**
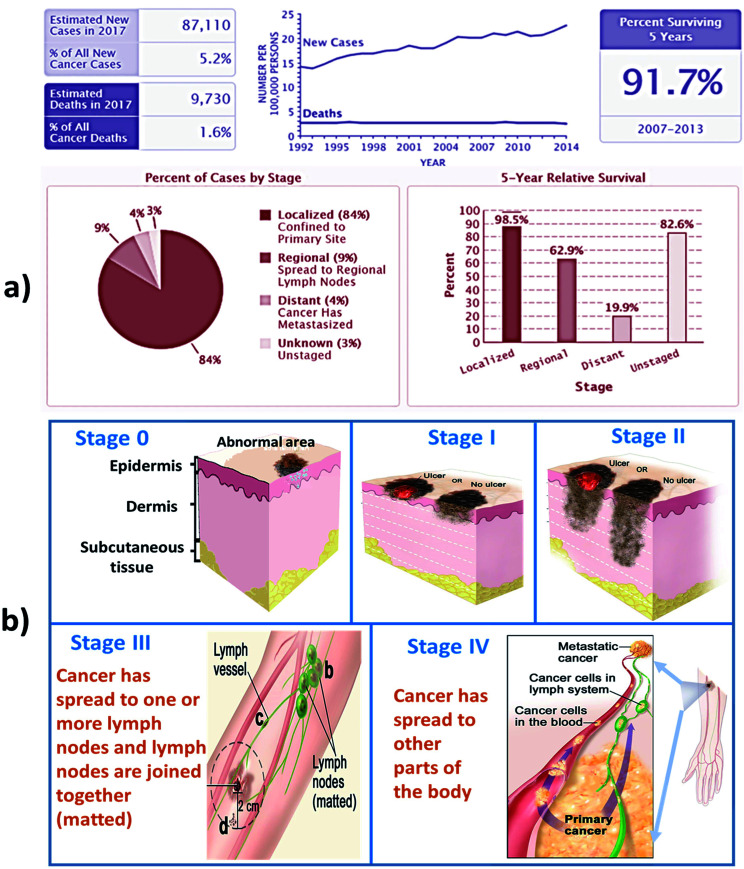
shows (a) recent stats and (b) stages of skin cancer. (Data Source: NCI SEER Cancer Stat Facts).^7^

A survey in 2015 found about 17.5 million cancer cases worldwide and 8.7 million deaths occurred due to cancer. As part of the epidemiological transition, cancer incidence is expected to increase in the future, considering limited health care resources. Past statistics and trends are shown in Fig. 1. In 2017, out of 5.4 million cases of non-melanoma skin cancer, only 3.3 million people were treated in the US. Over 87 110 new cases of melanoma will be diagnosed in the US, but estimation found that 9730 people will die of melanoma due to lack of healthcare resources. About 2 to 4% cases of people are suffering from melanoma skin cancer in India.^1^ In a report published ten years ago, skin cancer ranked 10th as the most common cancer in Malaysia and accounted for 2.6% of all cancer cases in the country. Melanoma is not very prevalent in Malaysia and occurred in only 5.4% of the patients who attended the dermatology clinic in Hospital Kuala Lumpur, but awareness among them is very less.^2^ At present, between two and three million non-melanoma skin cancers and 132 000 melanoma skin cancers happen globally every year. According to the recent research on skin cancer trends in Asia, it was found that skin cancer rates among the fairer-skinned were approximately three times higher than those who generally have darker complexions. Prevalence rates of skin cancer in Singapore increased from 1968 to 2006, especially among older Chinese.^3^

## Classifications of skin cancer

Skin cancer is categorized into three types which are basal cell skin cancer, squamous skin cancer and malignant skin cancer. The first two skin cancers are grouped together as non-melanoma skin cancers. The stages of skin cancer is shown in Fig. 1.^4^ The features and the treatment involved in each type of cancers are discussed below.

### Basal cell skin cancer

Basal cancer cell (BCC) is the least aggressive of non-melanoma skin cancer derived from keratinocytes that resemble epidermal basal cells.^5^ BCC appears like a flesh coloured pearl likes a bump or a pinkish patch of skin. BCC is due to skin exposure especially found in the sun exposed areas such as the face, head, neck, arms, legs, and abdomen. BCC can spread over the body and grows in nerves and bones. BCC is often known as non-melanoma cancer.^6^ BCC occurs in at least 2–3 millions of people per year. BCC has low death rate. It can be cured by simple treatment like radiation therapy and tropical medications such as fluorouracil.

### Squamous cell carcinoma

Squamous cancer cell (SCC) is the cancer of keratinocyte cells found on the outer surface of the skin. It appears like a red firm bumps scaly patches. It is caused due to over sun exposure. It is more commonly found in people who have pale light skin. It is mainly found in the regions like ear, face, neck, chest, and back. More than 1 million of people were affected due to SCC and 80 000 people died. In past three decades, there is huge increase in SCC among the US. The death rate of SCC is higher than BCC but comparatively lower than melanoma. BCC and SCC carry the ultraviolet radiation and damage the DNA directly. SCC results in ulceration and ends up with bleeding disorders. It should be treated at the earlier stage else it will grow in the dense amount in the body. SCC is characterized by proliferation of invasive squamous cells which are able to invade other tissues. It can be treated by photodynamic therapy, tropical chemotherapy, and curettage and electrodessication. SCC is the second common cancer in the world. It is not dangerous than that of melanoma.^4,8^

### Malignant melanoma

Melanoma arises from melanocytes, specialized pigmented cells that are found abundantly in basal layer of epidermis.^9^ Malignant melanoma consist of four major histological subtypes frequently reported namely superficial spreading melanoma (70%), nodular melanoma (15%), lentigo maligna melanoma (13%), and acral lentiginous melanoma (2–3%).^10^ Melanoma is usually observed as a dark spot on the epidermal (outer) layer of the skin. It is the fast-growing cancer cells that have the ability to spread over the surrounding tissue of the body. The malignant melanoma is caused due to UV radiation and damages the DNA indirectly. The free radical and the reactive oxygen species are the two leading causes of DNA damage. Research indicates that intentness of sunscreen components into the skin, conjugate with 60 min exposure to UV leads to the increase of free radical in the skin.^8^ Globally in 2012 malignant melanoma occurred among 232 000 people, and 55 000 people have died.^11^ Malignant melanoma is the deadliest cancer, but it can be cured at early stages of cancer. It is normally treated with chemotherapy, targeted therapy, and radiation therapy.

Detection of skin cancer in the early stage is useful in treating the condition very quickly. Detection systems pave the way for the diagnosis, but the result was not that precise comparing to biopsy test which is an invasive method. Along with the cost involved, the after-effects of biopsy include severe infectious problem around the punctured area. If the condition found to be more severe in the biopsy test, then surgery is performed, this adds to the cost along with the complexities. Other methods like radiotherapy, chemotherapy and drug therapy can be given, but the loss of hair and sittings often makes the patient to the worst condition. By taking into account all these points, many sensors are fabricated and designed to detect the skin cancer in a non-invasive manner, unlike biopsy. In this comprehensive review article, the recent non-invasive approaches involved in skin cancer detections are summarised.

## Literature search performed

**Fig. 2:**
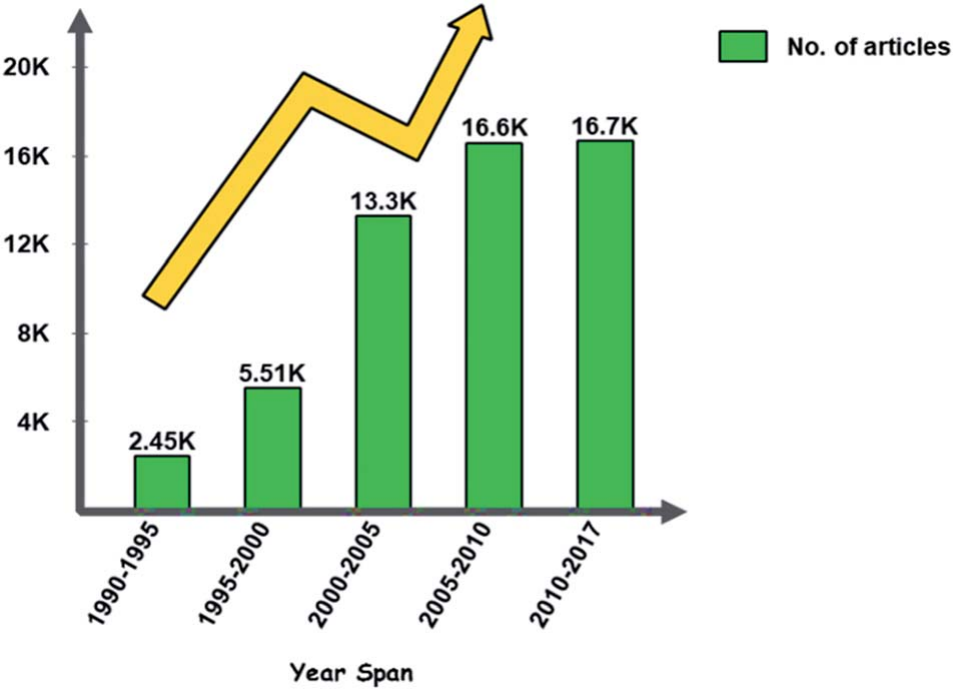
Publication trends over the years for skin cancer articles.

The literature search was performed using Google Scholar. The search was carried out for the keywords “Skin cancer, detection techniques, diagnosis, non-invasive” for the time span of 1990–2017. From the search, relevant articles were presented, and some articles were chosen based on the pertinence. Publication trends over the years are shown in Fig. 2. This review article will be promising for researchers who plan to explore further this field.

## Skin cancer detection and diagnosis

**Fig. 3:**
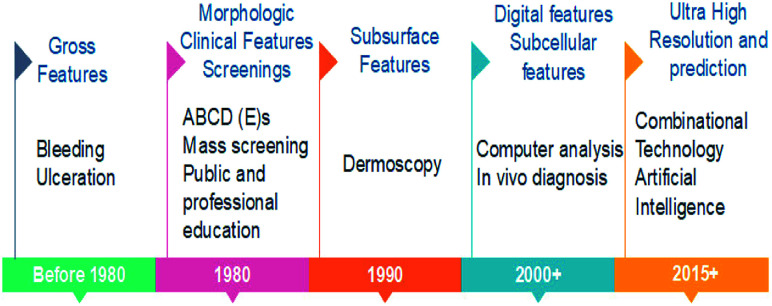
Roadmap of skin cancer diagnosis.

**Fig. 4:**
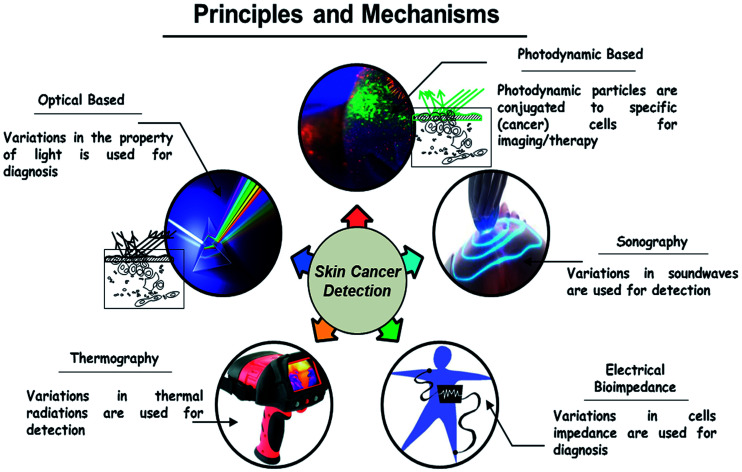
Principles and mechanisms for skin cancer detection.

Fig. 3 shows the road map of skin cancer diagnosis over the years. In this section briefly explained about the principle and detection mechanisms involved in skin cancer diagnosis as shown in Fig. 4. Along with that presented the currently practiced skin cancer diagnosis methods and techniques.^12^

### Principles and mechanisms involved

#### Optical

When the light is passed into the tissues, the light gets scattered within the tissues of the skin. The change in the property of the reflected light is used for diagnosis.^13^ Advanced biochemical markers are also developed with respect to cancerous tissue and specific wavelengths are used to enhance the contrast of the tissue by absorbing light.^14,15^

#### Photodynamic based

In order to detect the presence of tumor cells, a photosensitive marker is introduced into the particular area. Protoporphyrin IX is formed as a result of chemical reactions inside the body, which is absorbed by the healthy cells but gets accumulated in the tumour cells. When the resectoscope is introduced into the area, under blue illumination the accumulated pigment in the tumour cells emit red fluorescence and normal cells will be in blue. To provide contrast for photodynamic diagnosis (PDD) yellow-light filter is specially designed which enhances the red fluorescence. Skin lesions using photodynamic diagnosis are based on the fluorescent properties of an exogenous and endogenous compound in response to illumination. Stimulation of photosensitizers is made to produce the photodynamic effect when a certain wavelength of light is exposed to it.^12,16^

#### Sonography

Sound waves are used in this approach. After transmitting sound pulses into the body, some of the sound waves get reflected back to the probe, while some travel on farther until they arrive another boundary and get reflected. The reflected waves are sensed by the probe and relayed to the machine. The changes in the property of sound waves are used in this technique. Sonography is used to assess skin lesion depth and margins before doing the biopsy and also used to classify adjacent lymph nodes whether it has become cancerous or not. This is added to a patient's protocol to clarify lesion depth, identify margins for excision, and determine lymph node. It has the ability to differentiate benign from malignant skin cancer. It is used to identify metastatic lymph nodes visually, which avoids unnecessary removal of lymph nodes. The main advantage is that it gives the accurate results of measuring skin cancer lesion thickness.^17^

#### Electrical bio-impedance

The electrical impedance of skin, when measured by topical application of electrodes, is dominated by the high resistance of the stratum corneum. Changes in stratum corneum hydration or structure can be followed by electrical measurements at low frequency. Stratum corneum must be bypassed to measure the living epidermis and dermis. Direct contacts of microneedles on stratum corneum penetrate and measure the electrical activity underlying the skin.^18^

#### Thermal imaging

Thermography is based on the phenomenon that any object of a temperature above absolute zero emits electromagnetic radiation. There is a definite relationship between the surface of a body and the intensity and spectral composition of its emitted radiation. By determining its radiation intensity, the temperature of an object can thereby be determined in a non-contact way. The human skin is a thermal interface with the human environment. Temperature distribution on the skin will be emitted in the infrared band that is useful in imaging the thermal distribution. Infrared cameras are used in clinical medicine to map the thermal distribution in the human body. Stress is applied to the imaging region so that the liquid crystal plate directly over the body make useful in capturing the image. Thermal imaging can also be performed in a non-contact manner through the infrared camera of high sensitivity. Comparing to other techniques which have false negative reports at a high probability, thermography provides high accuracy.^19^

## Techniques and methods

### Photography

**Fig. 5:**
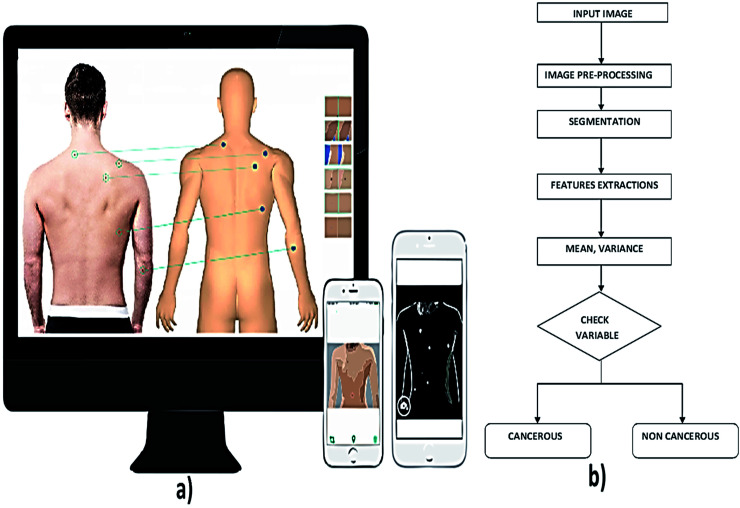
(a) Illustrates the total body imaging using photographic devices and (b) shows general steps in image processing technique for skin cancer diagnosis. Reproduced under CC license form ref. 43.

Total Body Photography (TBP) is also known as mole mapping which is used to identify patients with numerous nevi and changes in the lesions during regular skin examinations.^20^ TBP uses the digital camera for recording and storing the images as shown in Fig. 5. Using a high-resolution, professional medium-format camera, images are taken from the subjects by implementing the camera at different poses and position of the subject to get accurate results.^21^ Once the image is taken, it is digitally processed using mirror body mapping software. Sum of all the images is analyzed using this software. The software later generates an interactive, stand-alone program (DermaGraphix)^22^ containing the patient's body map. This stand-alone DermaGraphix body map, which is given to the patient using USB 2.0, tier 1 thumb drive.^23^ This system can also be performed at home by oneself, but the cost of equipment is high.^24^

Lynn T. Dengel, Gina R. Petroni and Craig L. Slingluff *et al.* had a significant finding of photography method in screening of skin cancer, but it is not used widely used due to time and cost.^20^ In this technique, captured the surface images of the skin to identify suspicious and pigmented lesions in high-risk patients primarily. Image processing algorithms are applied to the captured images for detecting any abnormalities in the skin. The segmentation technique which is used to detect the skin lesion is classified as region-based segmentation and neural edge detection. In region-based segmentation method, the optimal threshold is determined iteratively by an isodata algorithm. In neural network edge detection, an approximate closed elastic curve between the recognized neural network edge patterns is fitted.^25^

Computer-aided decision tools are essential in medical imaging for diagnosis and evaluation of various diseases.^26^ The acquired image from the subject using high-resolution camera is pre-processed to reduce the artefacts. To overcome the artefacts like the presence of hair in the image, mean, median, Gaussian and anisotropic filters are employed. It is followed by the detection of the lesion by image segmentation technique that partition the image into disjoint areas that are homogeneous concerning a chosen property such as luminance, colour, and texture. Next process is feature extraction.^27^ The extraction process takes by ABCD rule that checks the symmetrical, border, colour, and the diameter of the acquired image. Once the lesion is localized, different chromatic and morphological features can be quantified and used for classification.^28^ In the classification part, bringing out the algorithm in the examination process combines visual processing with deep learning.^29^ In deep learning, the computer is trained to figure out a problem rather than having the answers programmed into it.

The computer-aided technique is used to segment the melanocytes in the images taken from the skin. This segmentation is made mostly to reduce the local intensity variant. The mean-shift algorithm is adapted.^30^ The digital images of melanoma skin lesions can provide information about the skin cancer at an early stage without performing any skin biopsies.^31^ The mathematical morphology has been used for segmentation for proper identification of skin lesion area. Using TBP, different shape, texture and colour features are extracted and malignant melanomas have been classified sensitively.^32^ Delaunay triangulation is a technique used to extract a binary mask of the lesion region, which doesn't require any training. This is highly accurate when dealing with benign lesions, while the segmentation accuracy significantly decreases when melanoma images are processed. Geometrical and colour features were extracted from the binary masks generated by the algorithm for classification, which gives accurate results for melanoma detection.^33^

Computer-aided diagnosis extended its framework for the detection of skin cancer in recent years. The detection of melanoma skin cancer in the early stage will be very useful to cure it and safeguard the life of the affected individuals. Authors Abdul Jaleel, Sibi Salim, R. B. Aswin *et al.* found that based on imaging techniques and artificial intelligence the result of computer-aided detection of skin cancer is based.^34^ Computer vision plays a vital role in medical image diagnosis. Reported the computer-aided diagnosis for the pigmentations of skin lesions to determine the presence of skin cancer by performing the boundary detection.^35^ Skin lesions can also be analysed by qualification of degree of symmetry. The other paper explains that melanoma can be diagnosed by wavelet and texture analysis.^36^ The texture features were derived from the wavelet decomposition and the border features were collected from the lesions borders and the selections of such features were collected by using the gain-ratio method. That was computationally efficient for the melanoma diagnosis. In recent years, computer-aided diagnosis extended its framework in mobile technologies and in cloud platforms. By this method, the system can able to classify the lesions by identifying the moles in skin images and classify them accordingly as melanoma, benign and nevus lesions.^37^ Computer diagnosis can also be used as a preventive tool in the detection of skin cancer *via* mobile phone applications. By using this technique, the melanocytes can be separated from the histopathological images.^38^

Smartphone plays an active role in medical diagnostic of skin cancer by considering the sensitivity, specificity *etc.* The non-clinician users can easily differentiate the malignant and benign tumours.^39^ Through the computer-aided method, the patients can identify the skin cancer without going to the hospital.^40^ Computer-aided diagnosis can be carried out in the field of image processing, pre-processing, feature extraction, segmentation and classification of demographics images.^34^ Computer-aided melanoma diagnosis is used to provide the support to the physicians in enhancing the sensitivity and specificity in the diagnosis of melanoma condition. To capture the images of skin lesions, optoelectronic probe were used.^41^ One of the articles reveals that computer-aided diagnosis can be used to detect the early stage of melanoma by applying the image processing technique. Images can be analysed based on ABCD technique which involves asymmetry, border, colour, diameter *etc.* From the skin lesion images, the above parameters were collected by considering the texture, size and shape of the image.^26^ These features were extracted from the images and it was further processed for image segmentation. The extracted features were used to distinguish the normal skin and the melanoma cancer lesions as shown in Fig. 5b. The other methods deal with the image processing in which the images of the affected areas are captured by using the camera of the mobile phone by moving the diffraction grating. Thus the skin cancer can be diagnosed.^42^ Recent updates on photography techniques for skin cancer diagnosis are summarised in Table 1.

**Table 1:** Recent reports on photography based techniques for skin cancer diagnosis

S. no.	Description	Upside	Downside	Accuracy, sensitivity, specificity	Ref.
1	Optical threshold is determined in region based segmentation, whereas an approximate closed elastic curve between the recognized neural network edge patterns is determined in neural network edge detection	Best performance for lesions with a range of different border irregularity	Iterative thresholding technique is required	—	25
2	Different digital images have been analyzed based on unsupervised segmentation techniques followed by feature extraction techniques	—	Provides erroneous results if not all analysis carried out accordingly	Accuracy (Acc): 0.9748, sensitivity (Se): 0.8624, specificity (Sp): 0.9857	31
3	The mathematical morphology is used for segmenting the skin lesion in this method	Identification of skin lesion is clear	High cost and consume more time	Se: 85.71%	32
4	Delaunay triangulation is an accurate process for detecting the benign lesions, doesn't require training. Extraction of binary mask of the lesion is made	Accuracy level is high	Time consumption is more	Se: 93.5%, Sp: 87.1%	33
5	Polarized light is used for illumination and found that if plane of polarization is parallel then enhanced image is obtained if the plane of polarization is perpendicular then wrinkles or surface details disappear	Patient worry is reduced	Consumes more time	^—^	44
6	Smallest lesion of 1 mm is detected using photography technique and differentiation of melanoma and non-melanoma is detected using high resolution camera	Thinner melanomas are detected	Cost is high	^—^	24
7	Lower melanoma site region is detected using digital photography	Patient self-screening can be done	Cost is high	^—^	45
8	Pigmented lesion in the human body is detected using TBP which is useful in detecting the early stages of melanoma	Minute lesions can be differentiated using this technique	Instrumentation set up covers larger area	^—^	46
9	Incipient melanoma is detected using combination of both dermoscopy and photography in which accuracy of result is obtained	Not only detects the macroscopic changes and also the detection of dermatoscopic changes over time	Considerable proportion of melanomas misclassified as benign	^—^	47
10	Melanoma is detected in the initial growth stage itself with diameter of >6 mm and baseline images were obtained	—	Cost is high	^—^	48
11	Found that combination of total body examination, TBP and dermoscopy provides accurate result in detecting the early stage of melanoma	High resolution and sensitivity	Cost is high	^—^	49

### Dermoscopy

**Fig. 6:**
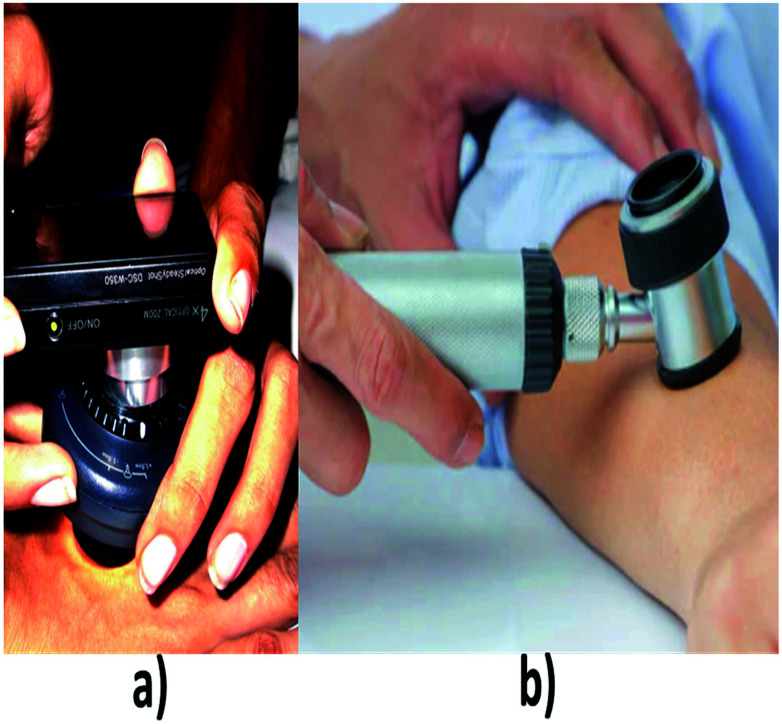
(a) and (b) shows different types of dermoscopes. (a) Reproduced under CC license from ref. 71.

Dermoscopy is also known as dermatoscopy or epiluminescence microscopy that is useful in examining the skin lesions.^50^ Conventional instrumental set up for dermatoscopy includes a magnifier (×10), a non-polarised light source, a transparent plate and a liquid medium between the device and the skin so that skin reflections can be avoided. Modern dermoscope as shown in Fig. 6, do not use this liquid medium; instead, it uses polarised light that eliminates the skin reflection.^51^ From this method, the non-cancerous and cancerous condition can be known. Basically, this dermoscope operates under three modes, first is, non-polarised in a contact manner, second is polarised light in a contact manner then the third mode is polarised light in a non-contact manner. The main advantage of using polarized light is, it provides better visualization in deeper skin structures whereas for non-polarized the information will be only on the superficial layer of skin.^52^ In this system, the accuracy level is increased based on the experience of the clinician.^53^

Dermoscopy shows subsurface structures of the skin with the use of handheld devices that extend optical light ray penetration beyond the skin surface and minimize surface reflection. In contrast, naked eye examination limits visualization of certain skin structures because the stratum corneum has reflective properties. Dermoscopy plays an important role in the diagnosis of skin cancer. It has higher discriminating power than a clinical analysis. The report made by Marie-Lise Bafounta, Alain Beauchet, Philippe Aegerter, on the diagnosis of melanoma skin cancer shown that sensitivity and the specificity^54^ obtained by dermoscopy techniques were comparatively higher than clinical analysis. Skin lesion on the face was usually diagnosed by comparing the dermoscopic features of lentigo senilis and lentigo malinga^55^ based on logistic regression method. Dermoscopy plays a crucial role in distinguishing the melanoma cells from benign cells with the help of various diagnostic tools^56^ such as pattern analysis, ABCD rule, Menzies method, and 7-point checklist. The dermoscopic studies were made on 20 pigmented skin lesions^57^ based on the pattern analysis and the results were found to be more accurate. Dermoscopy techniques are used to analyze the vascular structure^58^ of melanocytic and non-melanocytic tumors based on their morphological behaviour of the skin. Dermoscopy extends its application in early diagnosis of malignant melanoma and differential^59^ diagnosis of pigmented lesions of the skin by *in vivo* methods. The study was made on 2522 skin cancer subjects^60^ based upon the accuracy rate, dermoscopy techniques showed better results than conventional technique, but the main drawback of this technique is that it requires a high-resolution camera to capture the images of skin lesions.

Boundary detection technique which uses gradient vector flow for detecting the boundary is explained. The initialization method is automatic which makes the skin lesion border determination fully automated.^61^ The unsupervised approach for skin lesion images based on modified version of JSEG algorithm for detecting the border is used. The border detection error is quantified by a metric which uses manually determined borders by the dermatologist.^62^ An approach which is based on detecting the border using statistical region merging algorithm is reported. The border detection error is quantified by a metric which has three sets of dermatologist determined border as the base. This method is compared with four states of automated methods.^63^ A new method to detect and visualize the pigment network structures in the images already extracted has been reported. This detection is based on the automatic detection of the edges of pigment network structure which forms cyclic graphs. The extracted image is first pre-processed which gives the binary image; the binary image is then converted into graph and cyclic subgraphs which corresponds to the skin texture. These cyclic subgraphs are filtered to remove round structures.^64^ The mobile application uses the image processing technique for the prevention of melanoma. This system comprises of a microscope and a smartphone. The microscope is to image the area of skin, whereas smartphone is to get the output.^65^ The artefact is removed, and the borders of the lesion are detected by using border detection technique in the captured images.^39^

Dermoscopy also employed in the diagnosis of melanoma condition in pregnant women. The study was made on 35 pregnant women^66^ and the results showed that the pregnancy leads to significant modifications in pigmented skin lesions. Border detection^67^ method was used in dermoscopy techniques to enhance the accuracy level of the diagnosis of skin cancer. Mostly for non-melanoma^68^ skin cancer such as SCC and BCC are diagnosed by dermoscopic methods due to its high resolution. Using dermoscopy, the irregular streaks in melanoma can be detected by performing a three-way classification of streaks which involves regular, absent and irregular in a pigmented skin lesions.^69^ Esteva *et al.*, employed the deep convolutional neural network to classify melanoma and non-melanoma skin cancer.^70^ Latest reports on dermoscopy techniques for skin cancer diagnosis are summarised in Table 2.

**Table 2:** Recent reports on dermoscopy techniques for skin cancer diagnosis

S. no.	Description	Upside	Downside	Acc, Se, Sp	Ref.
1	Dermoscopy significantly has higher discriminating power than the clinical analysis. The sensitivity and the specificity ranges obtained from diagnosis of melanoma were found to be 0.75 to 0.96 and 0.79 to 0.98 respectively	More accurate than clinical examination for the diagnosis of melanoma in a pigmented skin lesion	Requires experience for better diagnosis	Se: 0.75–0.96 and Sp: 0.79–0.9	54
2	Compared the dermatoscopic features of lento senilis and lentigo malinga on the face by using logistic regression analysis	Analysis is easy	Resolution is low	Se: 93.8% and Sp: 52.3%	55
3	Dermoscopic images of the skin lesions were analyzed using 2-step diagnostic methods	Good computational capability	Various algorithms are needed	Se: 64.8% and Sp: 72.8%	56
4	Dermoscopic images of 20 pigmented skin lesions were evaluated based upon the menzie's method and ABCD rule and pattern analysis. It was found that results of pattern analysis were comparatively more accurate than any other methods	Web-based training is an effective tool for teaching dermoscopy	Involves training of practioners	Acc: 62.8%	57
5	The vascular structure of melanocytic and non-melanocytic skin tumours were evaluated based upon the morphological features	High resolution	Analysis of distinctive vascular structures is required	Se: 81.1%	58
6	Dermoscopy helps in early diagnosis of melanoma cancer by *in vivo* methods	Early diagnosis of melanoma	Resolution is low	Acc: 5% to 30%	59
7	Primary physicians made study on 2522 skin cancer subjects and the accuracy level were compared with the existing system	Improves the ability of physicians to triage lesions suggestive of skin cancer	Involves physician training, algorithm and expert consultation	Se: 54.1%, Sp: 71.3%	60
8	The studies were made on 35 healthy pregnant women and 35 age-matched female controls. The analysis showed that the pregnancy leads to significant modifications in PSL, especially with respect to globules, pigment network, and architectural order or disorder	Local intensity variant is done	Consumes more time to diagnosis the cancer	Se: 79.3%, Sp: 93.18%	66
9	In this method, the dermoscopic images obtained from the subject were analyzed based on border detection	Fast and accurate border detection	Requires a series of process algorithms	—	62
10	Through the dermosccpy technique the non-melanoma skin cancer were detected and the accuracy was high	Identification of skin lesion is clear	Only detects the non-melanoma cancer.	—	68
11	In dermoscopic images, the diagnosis of melanoma can be easily identified by irregular streaks	Accuracy level is high	Only includes the analysis of irregular streaks	Acc: 76.1%	69
12	Demonstrated the classification of skin lesions using a single deep convolutional neural network (CNN), trained end-to-end from images directly, using only pixels and disease labels as inputs	The CNN achieves performance on par with all tested experts	Involves CNN computing platform	Acc: 72.1 ± 0.9%	70

### Ultrasound

The ultrasound signal originates from the mechanical oscillations of many crystals in a transducer, which is excited by electrical pulses, this effect is called as the piezoelectric effect. Piezoelectric materials are ideal for this.^72^ The typical transducer consists of a disk-shaped piezoelectric element that is made vibrating by applying an electrical impulse *via* an electrode on each side of the disc. Likewise, the echo returning to the disk makes it vibrate, creating a small electrical potential across the same two electrodes that can be amplified and recorded.^73^ In modern clinical scanners, the transducer consists of hundreds of small piezoelectric elements arranged in a 1D array packed into a small enclosure.^74^ Thus the generated acoustic waves propagate in the fluid or surrounding tissues, from where these structures reflect the echoes which are collected back in the transducer which then transforms into electrical energy.^75^ This signal is then processed to get the image of the tumours in a required dimensional form. Photoacoustic imaging (PAI), or optoacoustic imaging, is insensitive to photon scattering in the biological tissue and, unlike conventional optical imaging techniques, this technique makes high-resolution optical visualization.^76–79^

**Fig. 7:**
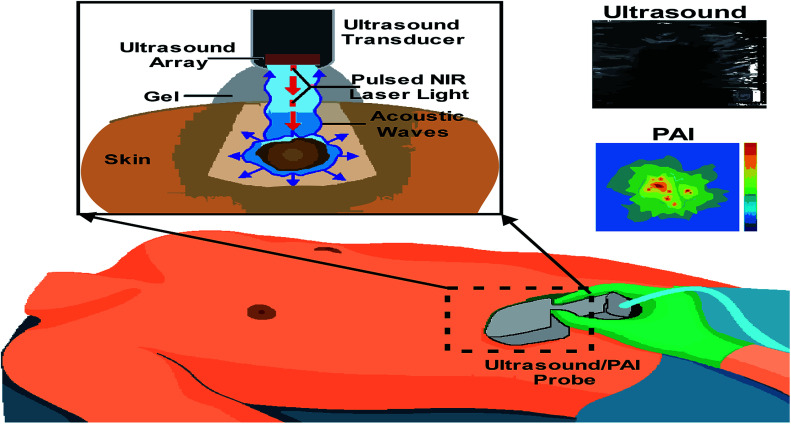
Integrated real-time photoacoustic/ultrasound imaging.

Multi-spectral optoacoustic tomography (MSOT) is an imaging method that produces high-resolution optical images in scattering media, including biological tissues. This technique is also known as functional photoacoustic tomography. MSOT illuminates tissue with light of transient energy, typically light pulses lasting 1–100 nanoseconds. The tissue absorbs the light pulses, and as a result undergoes thermo-elastic expansion, a phenomenon known as the optoacoustic or photoacoustic effect. This expansion gives rise to ultrasound waves (photo echoes) that are detected and formed into an image as shown in Fig. 7.^80–82^ The process is carried out with the help of computer where the image displayed on the screen is useful in diagnosing the lesions.^83^ The images are obtained in vertical sections are both the resolution and penetration vary according to the frequency. A retrospective study examined the usefulness of high-frequency ultrasound in the diagnosis of BCC through the identification of hyper-sonographic spots. This study concludes the conflicts between hyper-sonographic spots *versus* hypersonic spots in differentiating BCC and melanoma.^12^

Sonography is the pre-operative studies which measures the thickness of the tumor and internal echo density.^17,84^ Ultrasound of B-scan technique is interfaced with the computer which uses 20 MHz frequency transducer in acquiring the image.^74^ 40 MHz ultrasound gives higher resolution than 20 MHz.^85^ El Gammal *et al.* demonstrated the detection of melanoma using 100 MHz frequency which provided higher resolution and detailed visualization of skin layers.^86^ High-frequency sonography which is coupled with color Doppler gives 100% specificity and 100% sensitivity in detection of melanoma.^87,88^ Ultrasonography is used to produce 3-dimensional imaging and detects the border of the lesions.^89^ White light digital photography in combination with reflex transmission imaging, which is a form of high-resolution ultrasound used to classify the pigmented lesions.^90^ Contrast-enhanced ultrasound is used to study the primary lesion and gives information about the non-surgical treatment. Sonoelastography is used to study the mechanical behaviour of the skin lesions.^91,92^ Melanoma appears as hypoechoic or as heterogeneous oval structure under ultrasound detection.^93^ Detailed anatomic data about the primary tumor is provided by sonography.^94^ Retrospective analysis is done by ultrasound for pre-surgical examinations.^95^ Fine-needle aspiration cytology is also used as a minimally invasive technique of ultrasound in detecting melanoma.^96–98^ Recent summarization of ultrasound techniques for skin cancer diagnosis is presented in Table 3.

**Table 3:** Recent reports on ultrasound based techniques for skin cancer diagnosis

S. no.	Signal used	Description	Upside	Downside	Acc, Se, Sp	Ref.
1	20 MHz	Sonography with 20 MHz frequency is used as an pre-operative diagnosis of malignant melanoma	Hypo-echoic is identified easily with greater tumour thickness	Definite differential diagnosis is not possible	—	17
2	40 MHz	It is the superior, non-invasive method for the detection of BCC	High resolution	Microscopic examination is still required	—	85
3	7.5–20 MHz	The probe which is used has a high frequency which can detect the three layers of the skin	It is used to find the depth of melanoma	Resolution is less	—	84
4	100 MHz	It uses imaging technique which uses the transducer of frequency 95 MHz, whose resolution is high	Detailed visualization of the upper skin layers as compared with 20–40 MHz sonography	Image acquisition takes much time	Resolution (res): 8.5 μm	86
5	20 MHz	B-scanning ultrasound with 20 MHz frequency is interfaced to the computer and assessed acoustic shadowing and entry echo line enhancement for 29 basal cell papillomas (BCPs) and 25 melanomas	High-performance screening tool to assist in the discrimination between BCP	Not good in discriminating between benign naevi, and melanoma. Accuracy of detecting melanoma is not that accurate	Se: 100%, Sp: 93%	74
6	7.5 MHz	Fine-needle aspiration cytology procedures were performed in melanoma patients with palpable tumors or non-palpable, ultrasonically suspicious lesions. Device was interposed between needle and syringe which was vacuum valued	The procedure is painless and well tolerated by patients	Ultrasound guides the process and is minimally invasive	Se: 94.6% and Sp: 100.0%	96
7	7.5–15 MHz	Ultrasound B-scan is a technique used for detection of regional melanoma	Quick and reliable	Only in combination with cytologic examination of fine-needle aspirates, allows definite diagnosis	Se: 89.2%, Sp: 99.7%	97
8	13 and 20 MHz	Intralesional vascularization is assessed by the use of coupled high-frequency sonography and color Doppler	Simple and reliable.	Requires experienced operators	Se: 100% and Sp: 100%	87
9	Less than 15 MHz	Ultrasound is used as an imaging technique which uses transducer of superior designs	Parameters are quantified.	Photo damage occurs	—	83
10	7.5–10 MHz	Ultrasonography is used to produce a 3-dimensional size and outline of the lesions	Used to distinguish benign and malignant tumors	The quality of information depends heavily on the examiner's skill and experience	Se: 99.2%, Sp: 99.7%	89
11	7.5–13 MHz	Evaluated the distribution of metastases from scar of primary to the regional lymph node with subjects of cutaneous melanoma	—	Suitable only for follow up cases	—	99
12	20 MHz	Ultrasound technique distinguishes the nevi from melanoma using probes of different frequencies	—	Frequency of probes differs for various types of lesions	Se: 100%, Sp: 79%	90

13	20, 75, 100 MHz	Thickness of melanoma is assessed using different frequency in the transducer probe. Among all the frequency, using 7.5 MHz in transducer proves to be more versatile in detecting the thickness of melanoma	Information about the dermatology is provided completely	Sometimes may be overestimated due to infiltrate process	Se: 99%, Sp: 100%	88
14	10–100 MHz	Asymmetry of the tumour is determined by applying the gel on the skin surface and examining with the ultrasound with different frequencies	Deepest tissues are also analysed.	Pigments (melanin) of the skin cannot be detected	Se:92%	91
15	20–100 MHz	The sonographic findings in common benign and malignant skin tumors, inflammatory dermatologic diseases, and ungual and cosmetic conditions, among others, are considered and compared with various systems	Discrimination is better for different cutaneous layers	Visualization problem arises	—	93
16	15–22 MHz	Demonstrated use of sonography in facial BCC diagnosis. A comparative discussion was reported with other methods of assessing tumours and invasion besides sonography	Doesn't have any radiations	Provides only preliminary detection	—	94
17	20 MHz	High resolution ultrasound is used to detect metastases in melanoma subject by monitoring lymph nodes.	Early detection of recurrences can be made.	Not clinically used due to high cost	Se: 65%, Sp: 99%	98
18	20 MHz	Hyperechoic spots of BCC tumors are analysed using retrospective analysis of ultrasound examinations	Less expensive	Quality depends upon operator's skill	Se: 79%, Sp: 53%	95
19	7.5–100 MHz	Reported that sonography was effective at determining lesion thickness, defining lesion borders, and aids to identify whether lymph nodes are metastatic	Can be used to obtain measurements of skin cancer lesion thickness and can define lesion boarders. It can further delineate metastatic lymph nodes	Applicable only for screening method and not used widely by physicians due to lack of data's proving its accuracy level	Se: 90.74%, Sp: 98.89%	92

The laser transmits nanosecond pulses of NIR light through the transducer into the tissue. This light is then absorbed inside the tissue producing a localized temporary thermoelastic expansion. This expansion leads to the emission of ultrasound, which is detected by the transducer. Finally, a photoacoustic image is generated and displayed in real-time.

### Confocal microscopy

**Fig. 8:**
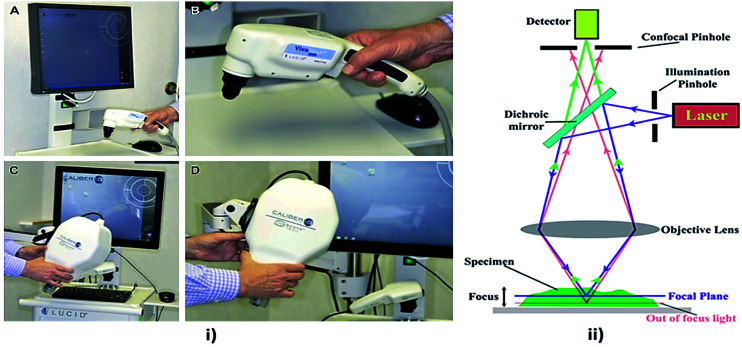
Confocal microscopy imaging technique. In image (i) (A and B) showing hand held RCM probes and (C and D) showing wide probe RCM, reprinted from ref. 110 with permission from Elsevier, and (ii) showing its mechanism, reproduced under CC license from ref. 111.

Confocal microscopy is a non-invasive technique that allows examination of the skin with cellular resolution. It uses a focused laser beam to illuminate a specific point within the skin and measures the reflection of light from that point as shown in Fig. 8. Multiple points are scanned across an area parallel to the skin surface to construct a grayscale image.^100^ Different depths can also be imaged to form optical sections. A confocal microscope includes a light source, a condenser, an objective lens and a detector. A confocal microscope uses point illumination and a pinhole in an optically conjugate plane in front of the detector to avoid out-of-focus signal – the name “confocal”.^101^ This configuration permits the collection of light from the single in-focus plane and dismissal of light from all out-of-focus planes. The light source illuminates a small 3-D spot (voxel) within a sample, such as skin, from which mirrored light is collected to produce a pixel.^102^ The illuminated spot is then scanned horizontally over a 2-D grid to obtain a horizontal microscopic section. This process is known as “optical sectioning” of the image in a series of horizontal planes stacked vertically to produce an image pixel by pixel, with an axial thickness of 2–5 μm.^103^ This property enables a confocal microscope to look at a slice in the body of a thick semi-transparent sample, whereas, conventional microscopes visualize all the planes contemporarily.

Confocal microscopy provides the capacity for direct, non-invasive, serial optical screening for thick, living specimens with least of sample preparation as well as a minimal improvement in lateral resolution, commonly called reflectance confocal microscopy (RCM). *In vivo* RCM is a non-invasive technique that allows examination of the skin with cellular resolution. Resolution is almost comparable to the conventional histology. It has the advantage of allowing the clinician to do a “virtual biopsy” of the skin and obtain diagnostic clues while minimizing unnecessary skin biopsies.^104^

Various skin disorders of which both hyper-pigmentary and hypo-pigmentary lesions can be detected using confocal microscopy.^105^ Also in combination with Raman spectroscopy at various wavelength, confocal microscopy provide better cellular details.^106^ The recent study involves the usage of vivascope^107^ with the extended version which offers better imaging. From 488 to 700 nm, the laser source can be used to illuminate the tissue non-invasively to know the depth nature of tissues and its complications.^108^ Multispectral polarized light imaging (MSPLI) and confocal microscopy combination provide better accuracy details in results rather an individual system.^109^ Recent progress in confocal microscopy techniques for skin cancer diagnosis is presented in Table 4.

**Table 4:** Recent updates on confocal microscopy techniques for skin cancer diagnosis

S. no.	Signal used	Description	Merits	Demerits	Acc, Se, Sp	Ref.
1	633 nm	Imaging is based on the detection of singly backscattered photons from the optical section and contrast is due to the relative variations in refractive indices and sizes of organelles and microstructures	RCM imaging with optical sectioning of 2–5 μm and resolution of 0.5–1.0 μm can be performed	Results vary according to the expertise in the clinical field	For melanoma, Se: 93% and Sp: 78% and for BCC, Se: 92% and Sp: 91%	112
2	820 nm	Light emitted by a laser diode (a safe wavelength for patient and operator) is reflected, directed through a small pinhole, and arrives at the detector, where an image is formed of horizontal sections of the different layers of an area of the skin depicting intracellular structures	Used in combination as complementary imaging tools, all those techniques will provide detailed, higher sensitivity, information about field cancerization, with increased specificity for higher accuracy of diagnosis and delivery of optimal treatment	High cost	—	101
3	450–650 nm	Testing the feasibility of a new mosaicing algorithm for perioperative RCM imaging of non-melanoma skin cancer margins on patients during Mohs micrographic surgery (MMS)	Video acquisition and video-mosaicing have improved RCM as an emerging tool	Cost is high	—	113
4	450 nm	Described dermoscopic and confocal features of 2 couples of similar lesions in order to achieve the correct diagnosis and the best therapeutic approach	Simple technique to implement	Difficult to diagnose lesions such as pink tumors	—	114
5	—	Reported that in three children RCM examination of cutaneous lesions of Langerhans cell histiocytosis showed specific signs correlated to histopathology	Aids in decision making for biopsy	Cost is high	—	115
6	—	Confocal microscopy a non-invasive tool for detection of various skin disorders such as melasma and vitiligo or melanocytic lesions such nevi and melanoma	Both hypopigmentary and hyperpigmentary disorders can be evaluated using confocal microscopy	Cost of equipment is expensive	—	105
7	720–950 nm	Development of integrated system of both confocal microscopy and Raman spectroscopy in the evaluation of melanoma	In depth microstructure analysis is performed using this microscopic technique	Integration of this system cost is high	—	106
8	830 nm	Multimodality imaging technique using high resolution microscope, different cellular level features are observed	Among the non-invasive techniques, reflectance confocal microscopy imaging is unique to provide cellular details	Difficulty in distinguishing dendritic melanocytes in pagetoid pattern from Langerhans cells	Se: 96.6% Sp: 89.2%	107
9	488 to 700 nm	Point source of light emitted by laser chamber falls on the tissue layer to provide the refractive property of normal and melanocytic skin	Real time imaging can be performed	Detection of tumor in the superficial layer with RCM is limited	Se: 97.3%, Sp: 72.3%	108
10	630, 660 and 830 nm	Combination of confocal microscopy and multispectral polarized light imaging (MSPLI) provide imaging in cellular level	Good correlation of result equal to histology	Small field of view	—	109

### Raman spectroscopy

**Fig. 9:**
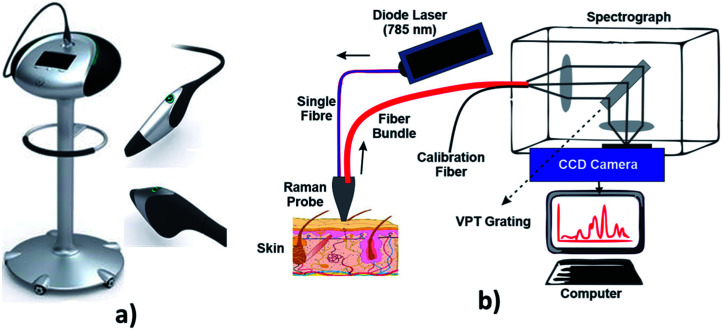
Raman spectroscopy technique. In image (a) showing hand held probe, (Courtesy of Verisante Technology Inc., Canada; with permission) and (b) showing the Raman spectroscopy mechanism.

Raman spectroscopy is a technique which is used to discover various modes in a system which involves vibrational, rotational and other low–frequency modes. The detailed setup of Raman spectroscopy is as shown in Fig. 9. It depends on Raman scattering of monochromatic radiations, usually from a laser in the visible, near infrared and near UV rays.^116^ In Raman scattering, inelastic collisions take place between the photons of an irradiating laser beam and the sample (or tissue) molecules.^117^ The obtained spectra can be processed and analyzed in order to provide automated feedback at the time of measurement.^118^ This system provides better sensitivity in differentiating the tissues.^119^

One of the article deals with the classification of skin lesion using Raman spectra by a non-linear neural classifier.^120^ The classification model is highly probabilistic and automated. This model involves the feature extraction for Raman spectra and a fully adaptive robust feed-forward neural classifier. The component analysis of Fourier transform Raman spectra of *ex vivo* basal carcinoma can be diagnosed by using the Nd:Yag laser at the wavelength of 1064 nm acting as an excited source in FT Raman RFS100/s spectrometer.^121^ Thirty-nine set of human skin samples were collected. Among the 39 samples, 18 samples were found to be histopathologically diagnosed as non-diseased and 21 samples were found to be BCC. Raman microspectroscopy is mostly done on *in vivo* method. It is a non-invasive, automated and real-time application to diagnosis the non-melanoma skin cancer which includes both BCC and SCC which can calibrate the sample within less than 1 second.^122^ By using the confocal Raman system^1^ with a handheld probe, the Raman spectra of 21 suspected non-melanoma skin cancers in 19 subjects with matched normal skin spectra were collected to analyse the pathological condition. Small distinctive bands corresponding to specified Raman spectra of lipids and proteins are used to distinguish the skin lesions. Raman Spectra of various compounds can be analysed by using the partial least regression and discriminate analysis.^123^ Raman spectroscopy plays a major role in the study of static and dynamic properties of the biologically significant molecule in solution, living cells and cell culture *etc.* The contribution of various bio-compounds such as lipids and proteins in the tissue can be estimated by providing a linear least square fitting model, to analysis the skin cancer in the tissue regions.^119^ Raman spectroscopy can also be used as a developmental tool in medical diagnosis. By shifting the excitation energy of Raman spectroscopy from the visible to NIR regions, the fluorescence components present within the normal cells can be minimized. The software tools have been developed to interpret the spectra of the tissue to diagnose the affected areas of the tissues regions.^124^ Cluster analysis plays a significant role in the classification of Raman spectra of malignant tumours from normal cells.^125^ Latest updates on Raman spectroscopy techniques for skin cancer diagnosis are summed in Table 5.

**Table 5:** Recent reports on Raman spectroscopy techniques for skin cancer diagnosis

S. no.	Signal used	Description	Merits	Demerits	Acc, Se, Sp	Ref.
1	1064 nm	Using the near infrared Raman spectroscopy, the molecular structures of normal and abnormal tissues are analysed	Skin lesions produced reproducible and unique spectra	Spectra are complex and an objective method for their interpretation remains to be developed	Acc: 94.8%	120
2	1064 nm	Near-infrared Fourier transform (NIR-FT) Raman spectroscopy was used to study the molecular alterations in the most common skin cancer, BCC	Can differentiate the normal cells from BCC	Needs neural network for classification	Acc: 95%	121
3	1064 nm	Raman spectra of normal tissues from benign and malignant can be differentiated by cluster analysis	Can differentiate the normal and abnormal tissues	Complex analysis is required	—	123
4	1064 nm	Nonlinear neural network is used to perform the Raman Spectra on the skin lesions to classify the cancer cell	The framework is highly automated	Complex algorithms are used	Acc: 80–95%	119
5	1064 nm	Basal carcinoma can be diagnosed by using the Nd:Yag laser at the wavelength of 1064 nm	This technique is applied to all types of tissues	Only analysis the non-melanoma cancer	Se: 83%	124
					Sp: 100%	
6	785 nm	Demonstrated an integrated real-time Raman spectroscopy system for skin evaluation and characterization, which combines customized hardware features and software implementation	Improved the signal-to-noise ratio by 16-fold	Involves a series of processing stages	Acc: 100%	125
7	825 nm	Demonstrated the capability of Raman micro spectroscopy to provide differential diagnosis of BCC, SCC, inflamed scar tissue, and normal tissue *in vivo*	High accuracy	Only diagnosis the non-melanoma skin cancer	Acc: 95%	126
8	1064 nm	Discriminate analysis and partial least square method is used to classify the cancer tissue	Good diagnostic accuracy	Detection needs optimum instrumentation setup	Se: 95–99%, Sp: 15–54%	116
9	830 nm	Linear least square fitting models is used to determine the biomolecules contributions	The distribution of lipids, protein, elastin, collagen compounds in normal and the abnormal tissues can be diagnosed	Samples becomes over heat due to high intense laser	Acc: 91.7% (actin *vs.* nucleic acid), Acc: 93.1% (collagen *vs.* elastin *vs.* melanin)	127

### Fluorescence spectroscopy

**Fig. 10:**
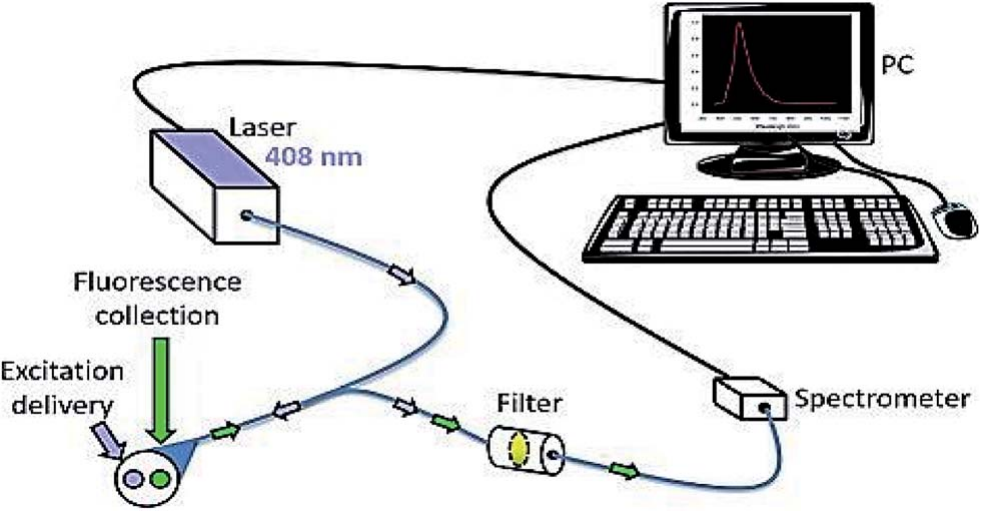
Fluorescence spectroscopy. It is used to detect the skin cancer with the help of fluorophores. Reprinted from ref. 145 with permission from Elsevier.

Fluorescence spectroscopy is electromagnetic spectroscopy also known as fluorometry or spectrofluorometry that analyses the fluorescence from the sample.^128^ The light source used in this technique excites the electrons in the molecules, and the fluorescence is analyzed from the samples as shown in Fig. 10.^129^ This method has been employed in many biological, biochemical and environmental applications.^130,131^ Laser-induced fluorescence spectroscopy is used to detect the skin cancer by *in vivo* methods.

A study was conducted by E. Borisova and his team on fluorescence spectroscopy; they noticed that excitation spectrum are usually identical to the absorption spectrum, as the fluorescence intensity is directly proportional to the absorption.^132^ The non-melanoma skin cancer can be diagnosed by the fluorescence polarization imaging technique, in which the tunable monochromatic light source along with CCD camera is employed to detect the tumour cells.^133^ As a result, two fluorophores were retained in tumours. Hyperspectral fluorescence is the one of the technique used to diagnosis the skin cancer in which the two ultrafast lasers were used operating at the wavelength of 355 nm that excites the auto-fluorescence between the bio-molecules that resides in the skin tissue.^134^ Auto-fluorescence technique is used to capture the images of the pigmented skin lesions, in which both the fluorescence and reflectance were studied.^135^ The excitation source used in this method is fibre coupled laser operated at the wavelength of 785 nm and the illumination of a light source is filtered by bandpass filter in order to acquire the fluorescence imaging and the reflectance imaging were obtained by the selection of components of the long pass filter. Auto-fluorescence technique is used to select the sampling points for the Raman spectroscopy, based on that the classification of various skin lesions was performed.^136^ Fluorescence spectroscopy is used to determine the distribution of biological substances by employing the fluorescent agent.^137^ The excitation source that causes fluorescence has been integrated with fiber optic probes to examine the tissue and it was recorded by using spectrograph. Fluorescence spectroscopy technique can also be used to classify the benign from malignant tumors with the help of fluorescence spectra by *in vivo* method. As a result, the normal tissues were found to be the wavelength of 440 nm and the non-melanoma tissues were found to be at the wavelength of 436 nm.^138^ Fluorescence spectroscopy has the ability to distinguish the cancerous cells from the non-cancerous cells.^139^ The monochromatic radiation is used as an excitation source at the wavelength of 300 nm. From the tissue two sets of fluorescence were emitted at the wavelength of 340 nm and 440 nm. The difference in intensity between the two fluorescence will decide whether the tissue is a malignant or a benign tumour.^140^

PDD of doubtful skin lesions relies on the fluorescent properties of an exogenous drug or endogenous compound in response to a light source. Topical agents are utilized to stimulate the reproduction of endogenous photosensitizers that produce a photodynamic effect when exposed to the light of specific wavelengths and energy. Photodynamic therapy (PDT) is the combination of absorption light and the photosensitizer.^141^ This technique also helps prior to surgery to detect the cancerous cells by using the photosensitizer. Fluorescence spectroscopy is performed on the tissues by using the photo sensitizer.^142^ Non-melanoma skin cancer can be diagnosed by fluorescence spectroscopy by both *in vivo* and *ex vivo* method. Auto-fluorescence is used to detect the presence of an abnormality in malignancy. The fluorescence intensity of dermal collagen is generally lower in tumour than in the normal tissues.^143^ Synchronous fluorescence method with time-resolved fluorescence technique can also be used for this applications.^144^ Modern reports on fluorescence spectroscopy methods for skin cancer diagnosis are summarised in Table 6.

**Table 6:** Recent reports on fluorescence spectroscopy techniques for skin cancer diagnosis

S. no.	Signal used	Description	Merits	Demerits	Acc, Se, Sp	Ref.
1	300 nm	The tumours are differentiated by the fluorescence intensity	High sensitivity	Samples become darker if proper dying agent is not provided	—	133
2	442 nm	Fluorescent agent is employed to analyze the bio molecule distribution	Distribution of bio molecule can be analyzed easily	Applicable only for detecting non-melanoma	Se: 96.6%	134
3	360–600 nm	Fluorescence spectra is used to classify the tumors at the wavelength of 436 nm and 440 nm	Short excitation source is enough	Less feasible	—	135
4	630 nm	Suppression of cytokine production is recorded	Cytokine production can be analysed	Two common side effects of PDT are cutaneous photosensitization and systemic immunosuppression	Acc: 83%	136
5	442 nm	A non-invasive diagnostic tool to identify diseased tissue and normal tissue from the absorption property of the chromophores	High resolution	Need high and optimum level of instrumentation setup	—	137
6	410 nm	Laser-induced fluorescence spectroscopies are used to detect the skin cancer by *in vivo* methods without biopsy	Can able to differentiate the normal tissue from malignant tissue	Highly engaged to external noise that disturbs the measurement	—	138
7	300 nm for epidermal tryptophan and 330 to 400 nm for dermal collagen crosslink	The fluorescence intensity of dermal collagen is generally lower in tumour than in the normal tissues	Abnormality in malignancy condition can be estimated easily	It requires endogenous fluorescence for diagnosis	—	139
8	650–750 nm	The tumours can be detected by using a tunable light source along with CCD camera	Fluorophore can be retained between the normal and tumour cells	High cost	—	140
9	355 nm and 440 nm	Two ultrafast lasers were used at 355 nm that excites the auto fluorescence	High resolution	External probe is required	—	141
10	785 nm	From the images of the pigmented skin lesions, both the fluorescence and reflectance were studied	High sensitivity	Cost is high	—	142
11	292 nm for tryptophan and 377 nm for collagen	The sampling points for Raman spectroscopy were chosen by auto fluorescence segmentation. It's function is to diagnose the BCC in sampled tissue during the Mohs micrographic surgery which is much faster than frozen section histopathology	Classification of skin lesions can be made easy	Segmentation is required to analyse whether the surgery is needed or not	Se: 100%, Sp: 92%	143
12	400 and 2400 nm	Cancer tissue can be diagnosed by synchronous fluorescence (SF) imaging	Highly efficient	It is a weekly emitting component.	Se: 82–97%	144

### Tetrahertz spectroscopy

**Fig. 11:**
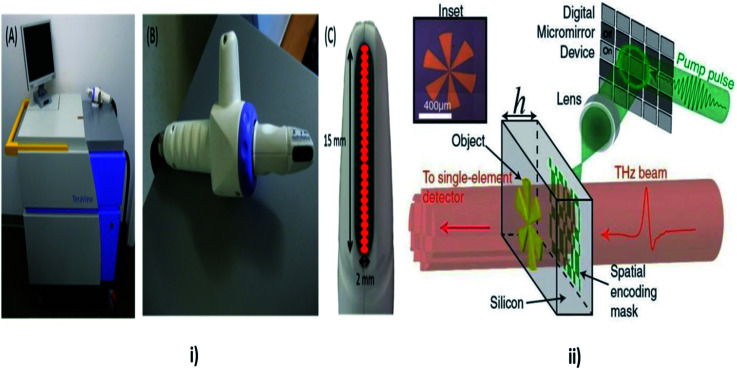
Tetrahertz Spectroscopy. In this technique the properties of matters are probed with short pulses of THz radiations. In the above image (i) showing TPI handheld probe system, (A) main unit with computer monitor, handheld imaging probe and black umbilical cord (visible on the right), (B) close up of the handheld imaging probe, (C) close up of the head of the imaging probe showing the black quartz window. The probe scans an area of 15 × 2 mm, and acquires data from 26 pixels (red), reproduced under CC license from ref. 156; and (ii) showing the THz mechanism, reproduced under CC license from ref. 157.

Tetrahertz (THz) spectroscopy as shown in Fig. 11 comes under the spectroscopy technique which is used to detect and controls the properties of matter with the electromagnetic domain that are in the frequency range between a few hundred GHz to several THz. It is an imaging technique, which is used to detect the epithelial cancers. Time domain analysis is used to differentiate between diseased and healthy tissues, which are significant statistically. The location of a tumour is well correlated and increased in terahertz absorption. Diagnosis of skin cancer using frequency domain analysis in terahertz spectroscopy is by using *in vitro* techniques and by using tetrahertz pulsed imaging (TPI). Then data manipulation is done to determine the spectral information which is related to depth.^146^

The pulsed THz spectrometry is used to analyse the dielectric properties of the human skin.^147^ Using this technique, the contrast between the BCC and normal healthy skin was found. The THz material parameter can encounter the dysplastic and non-dysplastic nevi pigmentation and it was compared with the normal healthy skin. A skin tissue model has been developed for the THz wavelength and has been estimated for a Monte Carlo simulation of the polarized light and scattering. The studies were made based upon the Mueller matrices in order to diagnosis the skin cancer.^148^ In accordance with the novel optic system, it is used to capture the images of the neoplastic and the non-neoplastic colon tissue with close to the wavelength – limited spatial resolution at the spatial resolution of 584 GHz frequency were employed to study the carcinoma tissues.^149^ THz technology plays an important role in medical diagnosis.

T waves also extends its application in both electrical and optical method to diagnose the skin cancer by means of plamonic photoconductive antenna of high sensitivity.^150,151^ THz spectroscopy also used to analyse the biological tissues by integrating the IR camera with IR detector.^152^ Using the THz imaging at the range of 1.39–1.63 THz the non-cancerous and cancerous cells can be differentiated.^153^ It also extended its application in the domain of biomedical which ranges from the bio-molecules includes lipids, proteins, amino acids to cells and tissues.^154^ It is used in sensing applications, involves detection of concealed objects, explosive detection of identifications, space exploration and THz cancer detections.^155^ Contemporary updates on THz spectroscopy approaches for skin cancer diagnosis are compiled in Table 7.

**Table 7:** Recent reports on Tetrahertz spectroscopy for skin cancer diagnosis

S. no.	Signal used	Description	Merits	Demerits	Ref.
1	0.1 to 2.5 THz	Dielectric properties of the human skin is analysed to differentiate the normal cell from abnormal cells	Acts as on effective tool to diagnose the skin neoplasm	Longer acquisition time	147
2	14 THz	Monte Carlo simulation and Mueller matrices are used to diagnosis the skin cancer based upon the polarization property of the skin	Sensitivity is high	Injection of nano particles is required	148
3	0.1–10 THz	Applied in the field of bio molecules of the cells and tissues	Cutting edge technology is addressed	It uses less lens array which results in less accuracy	154
4	0.1–2.5 THz	Involves the detection of tumours for skin cancer diagnosis	Sensing application can be done easily	It requires less lens array	155
5	0.3–4.3 THz	Used to image the neo plastic and the non-neo plastic colon tissue	Resolution is high	—	149
6	0.1–1.1 THz	Plasmonic photoconductive antenna uses the T wave to diagnose the skin cancer in both electrical and optical methods	Plasmonic photoconductive antenna element are available in single component with optical frequency	Cost is high	150
7	0.1–2.7 THz	Using the TPI technique the contrast between normal and tumour cells can be examined by time domain and frequency domain analysis	It requires only minimum impulse function	Consume more time	146
8	76 MHz	High sensitive of THz detection of nanoparticles can be provided by plasmonic photoconductive antenna	High resolution	High cost	151
9	4.2 THz	The biological tissue can be visualized by integrating the IR camera with the IR detector by exposing the human skin 4.2 THz	High resolution	Very expensive	152
10	1.39–1.63 THz	The contrast between the normal and cancerous cells are demonstrated using THz imaging technique	Easily differentiate the cells	Consume more time	153

### Optical coherence tomography

**Fig. 12:**
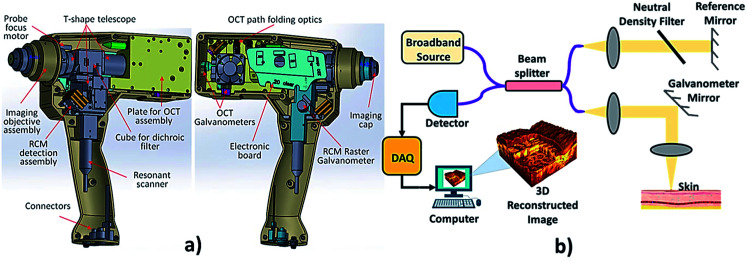
Optical coherence tomography. In the above image (a) showing a hand held OCT device, reproduced under CC license from ref. 176 and (b) showing the OCT mechanism.

The backscattering of near-infrared light by tissue is measured by the technique called optical coherence tomography (OCT) shown in Fig. 12. OCT is a non-invasive method that allows 2 or 3-dimensional cross-sectional visualization of the microstructural morphology of tissues.^158^ It provides better depth resolved image than ultrasound imaging. OCT is an *in vivo* technique which works on the principle of interference (Michelson interferometry) of infrared radiation and living tissues which is the major reason for high resolution. The interferometer in an OCT scanner splits a broadband source field into a reference field and sample field. The sample field focuses through the scanning optics and the objective lens to some point below the surface of the tissue. The back scattered beam from the tissue combines with the reflected beam from the reference mirror, forming an interference pattern which is detected by the photodetector.^159^ Structures of normal skin, including the epidermis, dermo-epidermal junction, dermis, hair follicles, blood vessels and sweat ducts can be clearly visualized by this method. This technique allows the differentiation between the benign and malignant epithelial tissue by the qualitative and quantitative assessment of OCT images. It enables the optical biopsy of epithelial lesions.^160^

A report was made by Michael R. Hee, Joseph A. Izatt, Joseph M. Jacobson, James G. Fujimoto, and Eric A. Swanson, who made a significant finding on OCT by introducing a *trans*-illuminance property in femtosecond laser.^161^ Famous authors Alexander M. Sergeev and Valentin M. Gelikonov found that longitudinal scanning of human tissue has good accuracy comparing to transverse scanning.^162^ Authors also used CCD camera to detect the photons but failed in showing the wider field of view.^163^ Cross-sectional image of human skin is obtained with higher accuracy.^164^ It has been proved that longer wavelength can minimize the influence of multiple scattering on image contrast and resolution; thus increase the effective penetration depth of OCT.^165^ Combination of both OCT and optical Doppler tomography (ODT) shows better results in scanning speed of acquiring the image.^166^ Even for detecting the inflammation on the skin, lesions on the skin and other infection over skin apart from tumour detection can also be performed by OCT.^167^ Heterogeneous property of tissue shows that collagen level in tissue can also rate the tumor growth.^168^ Later researchers like Aneesh Alex, Jessika Weingast and Martin Weinigel, reported that the combination of multi photon tomography (MPT) and OCT showed better resolution and high penetration depth.^169^ To overcome the conflicts in differentiating the non-tumour and tumour, deep margins are detected in the region of interest using the OCT.^170^ Increased thermal property of tissue shows various dysfunction of metabolic activity. The epidermal region shows higher thermal coefficient in the cancer patient.^171^ Also, the density of normal skin and tumour occurred in the skin varies.^172^ OCT reduces the Mohs micrographic surgery.^173^ Measurement of epithelial density can also be performed by OCT directly.^174^ Currently; precise tumour depth is performed by three methods combination namely, OCT, optoacoustic and Raman spectroscopy which shows the better results.^175^ Recent updates on OCT approaches for skin cancer diagnosis are compiled in Table 8.

**Table 8:** Recent reports on OCT techniques for skin cancer diagnosis

S. no.	Signal used	Description	Merits	Demerits	Acc, Se, Sp	Ref.
1	830 nm	With the help of fiber optic interferometer which releases a femtosecond pulses and coherent heterodyne detection to attain 130 dB dynamic range and time gating is performed to attain the image. Time gating is performed with the scattering medium	Monochromatic and coherent in nature	Cost is high	—	161
2	830 nm	A compactible OCT system is presented which uses fiber interferometer with integrated longitudinal scanning of tissue. The dynamic range 60 dB allows observing structure of human skin *in vivo* up to 1.5 mm in depth	Non-contact method	Consumes more time to analyse the structure	—	162
3	10 MHz	A slow-scan CCD-camera is able to detect very little numbers of photons. First measurements were made on models consisting of very small glass spheres embedded in polyester resin. It was possible to prove coherent photons from a depth of 2 mm	Accuracy is high in the obtained image	Consumes time since the measurement is performed with little number of photons so the field of view is less	—	163
4	830 nm	Based on the principle of low-coherence interferometry, cross-sectional images of the human skin can be obtained *in vivo* with a high spatial resolution of about 15 μm	*In vivo* investigation of pharmacologic effects	Detection depth is much lower	—	164
5	830 nm and 1285 nm	The effects of both instrumentation parameters and the dynamic characteristics of living tissue on image contrast and resolution and on speckle reduction are discussed. Both theoretical predictions and experimental results in human skin imaging show that longer wavelength can minimize the influence of multiple scattering on image contrast and resolution and thus increase the effective penetration depth of OCT	High resolution	Very expensive	—	165
6	1300 nm	OCT and ODT system that uses phase information derived from a Hilbert transformation to image blood flow in human skin with fast scanning speed and high velocity sensitivity. This technique decouples spatial resolution and velocity sensitivity in low images and increases imaging speed by more than 2 orders of magnitude	High scanning speed	Cost is high	—	166
7	1300 nm	A review was made on OCT method in a healthy skin, tumor region and inflammatory regions of skin and concluded that because of its non-invasive character, the technique allows monitoring of inflammatory diseases over time easily	Resolution is high	Data analysis takes times for investigating various skin disease conditions	—	167
8	1310 nm	Here polarization-sensitive OCT images of human skin *in vivo*, demonstrating the ability of the technique to visualize and quantify the birefringent properties of skin	Used to determine the polarization properties of the skin in real time	Results show poor categorization	—	168
9	1300 nm	Study demonstrates the clinical diagnostic potential of MPT/OCT for pre-screening relatively to obtain sub-cellular level information of the respective regions.	System design is simple and high speed image acquisition.	Cost is high	—	169
10	1318 nm	Deep margins of skin tumors are greatly assessed using OCT. Inflammatory skin diseases can also be detected	Differentiates premalignant from malignant lesions	Resolution is low	Sp: 83–100%, Se: 79–86%	170
11	1300 nm	The magnitude of the thermal coefficient of attenuation coefficient is greater in epidermis than in dermis. Thus it can be used to detect the optical attenuation using optical probe with the help of temperature control module	Can be used as a confirmation technique without physicians	Lack large scale clinical trials	—	171
12	1305 nm	While considering OCT base on density of the skin for detecting BCC, normal skin has higher densities whereas the lesion cells have lower densities than the normal skin	Non-contact approach	It requires much time for imaging	Se: 79–94%, Sp: 85–96%	172
13	—	To determine the margins of BCCs with OCT, prior to MMS (Mohs micrographic surgery), to reduce the number of surgical steps. Scans of the center and entire margin were performed. If parts of the BCC were visible outside the margin, another 2 mm were added and the scan was repeated until the tissue outside the labelling looked tumor free	Stages of MMS were reduced by OCT method	Expensive set up	—	173
14	1310 nm	To perform epithelialization of suction blister lesions by OCT and to find epidermal thickness (ET) easily as the primary outcome from histology test takes time. Results at earlier shows discrimination for neoepithelization but later shows better result	Fast speed scanning of image	This method not able to distinguish blood vessels from dilated lymphatic vessels. 20 experienced similar difficulties when evaluating OCT images of normal human skin	—	174
15	1300 nm	Combining OCT and optoacoustic modalities to provide precise tumor depth determination also with Raman spectroscopy to determine the tumor depth, volumetric imaging. Results found penetration depth is high in optoacoustic method	High resolution	Consume more time	—	175

### Multispectral imaging technique

**Fig. 13:**
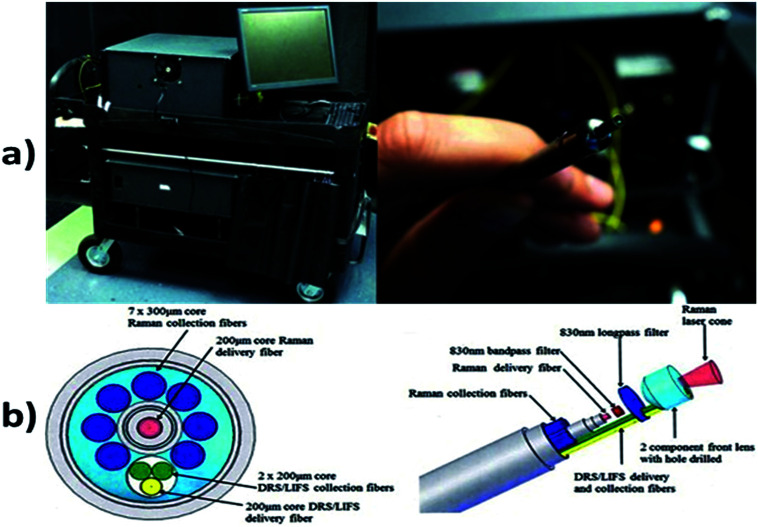
Multi-modal spectroscopy. In image (a) showing the setup of MMS and hand held probe and (b) showing exploded view with optical elements such as the filters and front lens identified along with the collection and delivery fibers for all three modalities. Reprinted from ref. 193 with the permission of AIP publishing.

In multi-modal spectroscopy (MMS) or multispectral imaging, various linear polarizers of different wavelengths are used. The spectral and spatial information of the samples are simultaneously recorded by the multispectral image spectrometer, in which the acquired images from a monochrome camera is processed using spectral and polarization filtering that provides high contrasting images which is useful in identifying the pathological and morphological features of the suspicious skin lesions.^177–179^ Refer to Fig. 13 shows the multimodal spectroscopy setup for skin cancer diagnosis. The report was made by Hagen Nathan, Kudenov Michael on multispectral imaging based on spectral bands. In this, the image analysis is done automatically and pattern recognition is used to identify lesions which in turns help in further biopsy. It plays an important role in the diagnosis of skin cancer by considering the parameters such as texture, asymmetry, border irregularities *etc.*^180^ The images are obtained from the affected regions of the skin using the charge coupled camera along with eight narrow band filters ranging from 450 nm to 800 nm at the interval of 50 nm.^181^ The features are extracted from the image using the principal components analysis. The characterizations of malignant and benign tumors are separated by spatial gray level co-occurrence matrix.^182^

Multispectral imaging technique includes *in vivo* methods in which the images were analyzed at the wavelength between 483 nm and 950 nm at equal wavelength intervals.^183^ Multispectral imaging employs the automated diagnosis of skin cancer using the neural network classifier. The neural network has the ability to discriminate the malignant and benign tumor with the sensitivity of 80.4%. In the multispectral imaging system, the pigmented skin lesions are separated by using the automatic segmentation algorithm.^184^ Usually, in multispectral imaging technique, the images of the skin lesions are analyzed as 2-dimensional and 3-dimensional images at the wavelength range from visible to the infrared spectrum.^185^ Multispectral imaging technique facilitates the analysis of skin lesions, vascular depth and sub-cellular pigmentations.^76^ It uses the radiometric measurement to analysis the malignant and benign tumors.^186^ A multispectral imaging technique employs the multispectral camera to capture the skin lesions images. The images are analyzed based on the spectral ranges between 450 nm to 950 nm which is used to discriminate the melanoma from nevus cells.^187^ The self-developed software is used in multispectral imaging technique to analyze the skin chromophores which is a non-contact method.^188^ Multispectral imaging method finds its applications in the detection of melanoma skin cancer by considering the parameters such as melanin index and erythema index.^189^ It also provides the decision of undergoing biopsy from the pigmented lesion using multispectral digital skin lesion analysis (MSDSLA) device.^190^ Multispectral imaging technique enhances the diagnosis by analyzing the vascular depth of the skin lesion.^191^ The vascular depth can be interpreted with the help of 6 layered skin models. Reported the multispectral imaging techniques combined with 3-dimensional imaging sensor to detect the skin cancer.^192^ Contemporary reports on MMS methods for skin cancer diagnosis are summarised in Table 9.

**Table 9:** Recent reports on multispectral imaging technique for skin cancer diagnosis

S. no.	Signal used	Description	Merits	Demerits	Acc, Se, Sp	Ref.
1	430–950 nm	In this technique the skin tissue are characterized by texture, asymmetry, blotchiness and boarder	Easily differentiate the malignant and benign tumors	High cost	Se: 100%, Sp: 97%	181
2	430–950 nm	The images of the moles are collected using charge coupled camera and the classifications of tumors are made by spatial gray co-occurrence matrix	Have high potential to classify the skin cancer	Texture information is needed	—	182
3	483–950 nm	The skin lesions were imaged by *in vivo* imaging system operated at the wavelength between 483 nm and 959 nm at the equal wavelength intervals	High sensitivity	Classifier is required.	Se: 80.4% Sp: 75.6%	183
4	483–951 nm	The pigmented lesions are categorized by automatic segmentation algorithm	Applicable only for non-melanoma skin cancer	Cost is high	Acc: 97.1%	184
5	405–910 nm	In this method, the 2-dimensional and 3-dimensional images were analyzed at the wavelength ranges from visible to infrared spectrum	High resolution	Only for the particular wavelength results were optimum	—	185
6	510–610 nm	In this method, the multispectral nevoscope trans illumination technique is used to compare the radiometric measurement of malignant and benign tumors	Differentiate the malignant and benign tumor	Vascular depth of the skin is hard to interpret	—	186
7	450–950 nm	Using the multi spectral camera the images of the skin lesions were captured and analyzed based upon the spectral ranges	Novel image processing algorithm to differentiate of melanoma from pigmented nevi	High cost	—	187
8	450–950 nm	The clinical trials were made on 266 pigmented lesions and 49 vascular lesions. The software system was used to mapping the skin chromophores	High clinical information from the image	Various algorithms are required for mapping process	—	188
9	450–950 nm	The melanoma images were analyzed based upon the melanin index and erythema index	High resolution images are obtained	Cost of instrumentation set up is high	—	189
10	400–720 nm and 650–1100 nm	MSDLA device is used to determine whether the biopsy is required or not	High resolution	High cost	Se: 94%	190
11	400–720 nm and 650–1100 nm	The six layered skin model was made to analyze the optical parameters	Accuracy level is high.	Doesn't classify the melanoma and non-melanoma skin cancer	—	191
12	414–995 nm	The skin cancer can be detected based on the spectral parameters which involve the reflectance and the color of skin lesions	High quality	High cost	Se: 93%, Sp: 54%	192

### Thermography

**Fig. 14:**
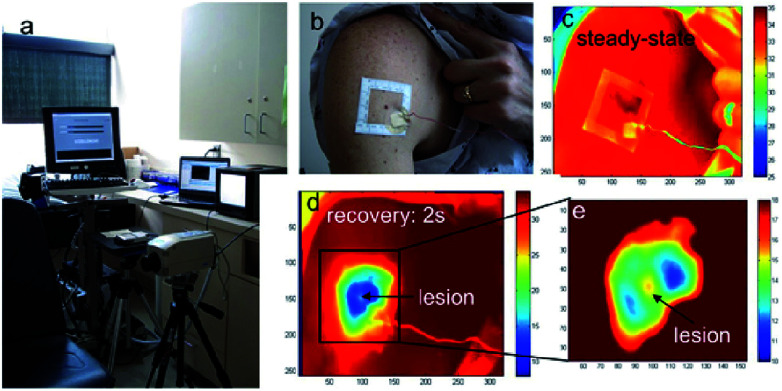
(a) The infrared imaging system, (b) photograph of the body surface area with a cluster of pigmented lesions, (c) reference infrared image of the region at ambient temperature, (d) the same area after cooling and (e) magnified section of the melanoma lesion and surroundings. Reproduced under CC license from ref. 19.

Infrared radiation is emitted by every object which possesses the temperature absolute zero point.^194^ The thermal imager determines the temperature of the object's surface based on the intensity of infrared radiation making it visible to the human eye with the thermal image. This process is referred as thermography. Thermal imager translates the wavelength from the infrared to the wavelength which is visible to human eye as shown in Fig. 14.^195^ This is the principle which is used to detect the skin cancer using thermography.^196^ Thermography has been employed in medicine for various applications.^197^ However, thermography overcomes all the shortcomings that other methods had.

**Fig. 15:**
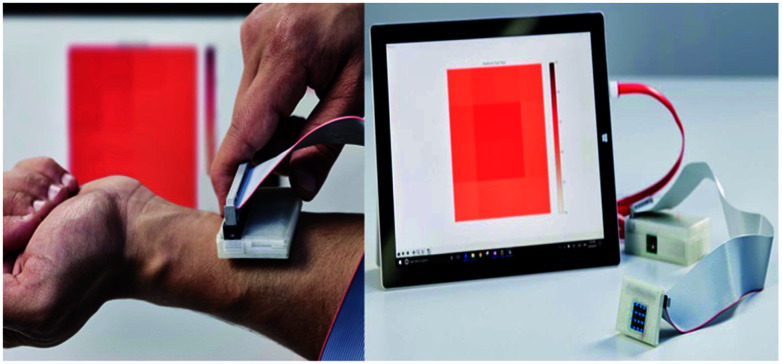
sKan device. This device detects skin cancer using thermal images that are obtained from affected areas of the skin surface using thermistors. Reproduced from ref. ^202^.

A study performed by Cila Herman^198^ found a significant finding on recently introduced quantitative dynamic infrared system QUAINT which measures differences in the infrared emission between healthy tissue and lesion during the thermal recovery process after removal of cooling stress. Thermistors, thermocouple and other advanced sensors are used to detect the variation in temperature.^199^ The rise in temperature is a significant symptom in the skin cancer patient. Nano type sensors are involved in detecting the changes in the biological analyses and the obtained information is processed to the local server in the hospital which is directed to the medical professionals in the hospital.^200,201^ Prateek Mathur *et al.* studied on temperature variation of thermal images obtained from skin cancer patient and introduced a device named sKan as shown in Fig. 15. It is a new technique which maps the affected area with the help of sensor and monitors the patient's stage in the skin cancer.^202,203^ This is achieved by having a template of temperature values on which the obtained value is mapped and the data are sent to the doctor for the analysis.^201^ Viktor Gruev *et al.* made a report on an imaging sensor capable of recording the optical properties of partially polarized light by monolithically integrating aluminium nanowire optical filters with a CCD imaging array. The imaging sensor consists of 1000 by 1000 imaging elements with 7.4 μm pixel pitch. The polarization imaging sensor captures intensity, angle, and degree of linear polarization in the visible spectrum.^204^ Current updates on thermography techniques for skin cancer diagnosis are presented in Table 10.

**Table 10:** Recent reports on thermography based techniques for skin cancer diagnosis

S. no	Device used	Description	Merits	Demerits	Acc, Se, Sp	Ref.
1	Image sensing device 340–380 nm	Using image sensing device illumination of ultraviolet light in the wavelength range of 340–380 nm, fluorescence property of the different skin anomalies were detected	Real time recording can be done and non-invasive	Highly expensive	—	205
2	Infrared camera	Infrared imaging takes place in different region of the tissues, which is caused due to micro calcification or calcification in the benign lesion	High resolution	Skin temperature varies for external noise that affects the result	—	206
3	Infrared thermometer	Thermal sensor that detect the affected area in a thermal map and calibrated using the processor (30) and corresponding output signals taken over a predetermined range of ambient temperatures	High resolution	Cost is high	—	207
4	Infrared detectors	Temperature distribution of the body surface is performed using infrared thermography which produces thermogram that further used for diagnosing procedure	High resolution	Emission from other surfaces affects the measurement accuracy	—	208
5	Amber radiance 1 T IR camera	Differentiation of melanoma and benign cutaneous lesions is performed using infrared thermography and found the sensitivity and specificity for different depths of the lesion	Real time recording and non-invasive	Need optimal instrumentation setup	Se: 39% Sp: 100% (0–5 mm lesions), Se: 58% Sp: 98% (lesions >5–15 mm), Se: 95% Sp: 100% (lesions >15–30 mm), Se: 78% Sp: 89% (lesions >30 mm)	209
6	Infrared thermal camera operated at 3 μm −5 μm	Thermal responses for healthy and malignant tissue were compared and concluded that increased metabolic activity of melanoma lesion can be detected using infrared imaging	High resolution	Camera has only 2% of accuracy	—	19
7	Infrared thermal camera operated at 14 000 nm	Symptoms of illness are detected using embedded devices which can be in the form of watch, glass, camera *etc.* Communication interface is attached to the embedded device so that the captured data is transferred into the local server of the hospital or other healthcare station	Automatically the captured data's are transmitted.	Cost is high.	—	203
8	Infrared camera	Thermal imaging for early stage detection of cutaneous melanoma is performed and resulting surface temperature oscillations were recorded using infrared camera	Real time recording	Lower rate transmission	—	210
9	Electrochemical sensor, optical sensor	Biosensors are used to detect specific biological analyze later by converting a biological entity into an electrical signal and analyzed. Biosensor has vast potential in detecting the cancer cells	Have more resolution comparing to other application sensor	Specific chemical analyzer is random in nature so difficult to interpret	—	201
10	Nd:YAG laser at 532 nm	The imaging sensor is use to capture the affected area which has 7.4 μm pixel pitch, composed of 1000 by 1000 imaging elements with a nanowire optical filter	The signal to noise ratio is high and consume less power	Cost is high since nanowire fabrication is implemented	—	204
11	Anon contact IR temperature sensor at 8–14 μm	The emitted IR range extends from 800 nm to few hundred micrometers which are detected for the temperature variation	Mapping of temperature variation is perfect	Resolution is less	—	211
12	Infrared detectors	The surface the sample is sensed with the small change in the motion. This motion is detected by bending of cantilever in the sensor system. Such system provides information about the position of the subject and the temperature value during displacement	Nanoparticles are synthesized in the sensor which makes the accuracy to be good	Cost is high	—	212
13	IR absorbance sensor at 3300 nm and 3570 nm	Infrared absorbance sensor used to detect the malignant cells in the biopsy. It takes 8 seconds to identify whether it is tumor or normal cell	This sensor is used in diagnosing the melanoma during histopathological analysis and also decreases the wrong interpretation	Supports shorter range and its performance degrade with longer distances	—	213

### Electrical bio-impedance

The living cells membrane act as an electrochemical membrane, which is a semi-permeable membrane allows only certain entities to pass through and does not allows the certain size of entities to pass through. The leaky capacitor acts as a semi-permeable membrane. It mainly consists of electrolytes and resistive properties. Biological tissues and cell suspensions have both capacitive and resistive properties. The impedance of biological tissues is highly frequency dependent. They travel in the extracellular due to high capacitance, high-frequency currents. Study of electrical bio-impedance of tumors was done way back in 1980's by Morimoto *et al.*^214,215^

**Fig. 16:**
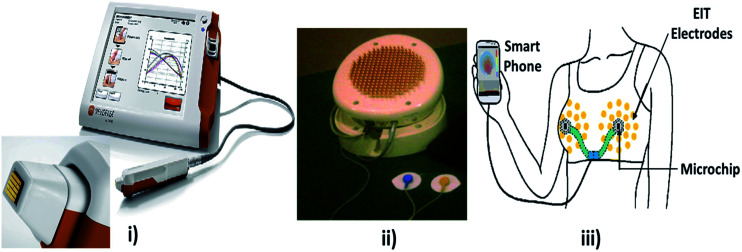
Electrical bio-impedance measurement setup. In the above image shown (i) Nevisense device including hand piece and touch screen monitor. (Courtesy of SciBase, Stockholm, Sweden; with permission); (ii) Electrical impedance mammograph meik, reproduced under CC license from ref. 227; (iii) Smart phone based diagnosis using EIT patch.

Electrical impedance tomography (EIT)^216^ is being used for various biomedical applications. A report given by Beetner D. G. *et al.* shows the difference in electrical impedance with the frequency range of 1 KHz to 1 MHz for the lesion which has a diameter of 2–15 mm.^217^ The measurement was done by using electrodes of a two-point measurement.^218^ The measurement of electrical impedance shows the morphological changes which relate to the growth of skin cancer lesion.^219^ The multifrequency impedance spectra are used to detect the electrical bio-impedance for different skin lesions.^220,221^ The non-invasive probe separates the nevi from BCC with 96% sensitivity and 86% specificity.^222^ The impedance is measured by use of impedance spectrometer between 1 kHz and 1 MHz of various types of skin cancers including melanoma. This improves the signal to noise ratio. By applying small alternating voltage and comparing the measured current with voltage, the impedance between two electrodes is measured.^218^ The change in impedance is used to detect the skin cancer by using the information about cell shape, structure and orientation, integrity of cell membranes, relative properties of intra and extra-cellular fluids and ionic composition. This device consists of the system on chip impedance converter, AD5933, an ultra-low power MCU, MSP430F6638, and electrodes. The electrode used is Ag/AgCl electrodes, which is non-toxic and low cost.^18^ A smart phone based early cancer detection using EIT patch electrodes has been demonstrated.^223^ Electrical impedance spectroscopy (EIS) algorithm is used in detecting the lesions which have high sensitivity in the detection of malignant melanoma.^224^ Electrical impedance helps in differentiating the cancerous cell from non-cancerous cell at the range of 1 KHz to 2.5 MHz.^225^ Bio-impedance spectroscopy also available in portable form which is used to monitor the physiological system.^226^ Latest progress on electrical bio-impedance approaches for skin cancer diagnosis are compiled in Table 11. Fig. 16 shows the different devices used for electrical bio-impedance measurement.

**Table 11:** Recent reports on electrical bio-impedance techniques for skin cancer diagnosis

S. no.	Signal used	Description	Merits	Demerits	Acc, Se, Sp	Ref.
1	1 kHz to 1 MHz	Used to distinguish BCC from benign lesions based on magnitude, phase, real part and imaginary part of the impedance calculated	It provides the rapid differentiation of tumors	High cost	Se: 90%, Sp: 50%	217
2	2 MHz with a sampling rate of 50 Hz	To determine the state of accuracy to distinguish benign from malignant lesions by measuring with electrical impedance	Reflects morphological changes when there is a growth in tumor cells	This method is not applicable for humans	Se: 92%, Sp: 67%	219
3	1 kHz and 1000 kHz	Distinguishes the skin cancer from the benign lesions using multi-frequency impedance spectra	The result obtained is better accurate than conventional methods	Distinguishing the tumors takes more time and false result may also be obtained	Se: 75–87%, Sp: 100%	220
4	1–1000 kHz	Compares the detection of skin cancer by non-invasive probe and micro invasive electrode system, whose surface is furnished with tiny spikes which gets penetrated to the stratum corneum	Electrode system produces better result	Minimally invasive technique	Se: 92–96%, Sp: 80–86%	222
5	1 kHz and 1 MHz	Describes the method for detecting the skin cancer using electric impedance. Electric impedance of the biological system decreases with the increase in frequency	High resolution	Multivariate and the impedance is complex	—	218
6	1 kHz to 2.5 MHz	Accuracy of electrical impedance to classify malignant melanoma from benign tumour by automated classification algorithm	Accuracy is high	Various algorithm is needed for classification of skin cancer	Se: 95%, Sp: 49%	221
7	1–100 kHz	Non-invasive approach for detecting the presence of skin lesions by measuring the impedance change	Low cost and portable	Electrodes are used which cause discomforts	—	18
8	1 kHz to 2.5 MHz	EIS algorithm is used on lesions to differentiate normal skin from the abnormal lesions	High resolution	Experienced physician is required	Se: 100% (non-melanoma cancer)	224
9	1 kHz to 2.5 MHz	1300 lesions were collected from multi centre and examined by EIS at 1 kHz to 2.5 MHz in order to differentiate the melanoma from non-melanoma cancer	Classification is easy	High cost	Se: 98.1% (melanoma), Se: 100% (non-melanoma)	225
10	20 kHz to 1 MHz	Portable bio-impedance system is used to diagnose the skin cancer based upon the magnitude ratio and phase detection method.	Act as a great tool for monitoring the physiological conditions of the biological system	High cost	—	226

### Tape stripping

**Fig. 17:**
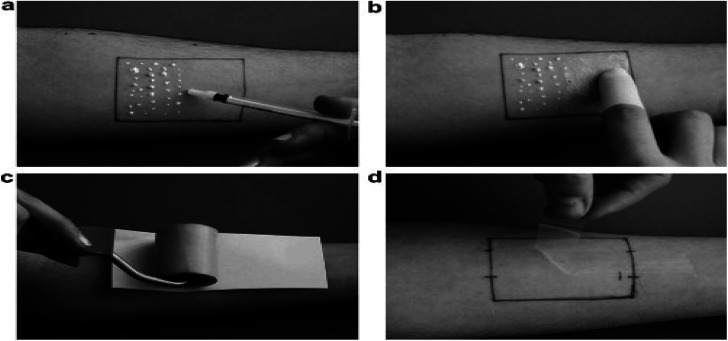
Tape stripping technique. Method of tape stripping (a) application of the formulation on a marked skin area; (b) homogeneous distribution; (c) the adhesive tape is pressed with a roller on the skin, an empty sheet of paper avoids the transfer of the formulation onto the back side of the tape; (d) removal of the tape. Reprinted from ref. 228, with permission from Elsevier.

Tape stripping is the removal stratum corneum layers as demonstrated in Fig. 17. Adhesive tape is used to remove the cell layers of the stratum cornea from the skin area. The number of corneocytes and a considerable amount of penetrated formulation will be present in the tape strips.^228^ Tape stripping is also known as Epidermal Genetic Information Retrieval (EGIR).^90^ The sample cells from the stratum corneum by the use of adhesive tape stripping from which RNA is isolated, amplified and gene expression is noted.^229^ This adhesive tape has the RNA genetic expression profile which is useful in differentiating melanoma from nevi.^230^ With the help of 17-gene genomic biomarker, the presence of melanoma is detected from the harvested specimens. This analysis is carried out using ribonuclease protection assay (RPA). This analysis is used to differentiate malignancies on the basis of gene expression profiles. DermTech's Epidermal Genetic Information Retrieval (EGIRTM) uses a custom adhesives film to collect surface skin samples. This method is painless and it can also be virtually applied to the skin surface. Also, this method has 100% sensitivity and 88% of specificity.^231,232^ It is a rapid and convenient method^233^ to contrast various changes in the cancerous patients. It also differentiate lentigo maligna melanoma from solar lentigo, which is sometimes difficult with histology.^234^ This method is widely used as a robust bedside system for subject with numerous dysplastic nevi.^235^ Can be performed from the extracted RNA only by freezing it up to −80 ^°^C. Only then the frozen sample is treated.^236^ Early detection can be achieved if tape stripping toluidine blue (TSTB)^237^ is combined with ABCDE rule. In 2011, Wachsman and colleagues showed that EGIR performed on 202 melanocytic lesions identified 312 genes that were capable of differentiating normal skin, nevi and melanoma.^229^ Recent progress on tape stripping techniques for skin cancer diagnosis is compiled in Table 12.

**Table 12:** Recent reports on tape stripping methods

S. no	Description	Merits	Demerits	Acc, Se, Sp	Ref.
1	mRNA from the cells attached with tapes are collected for gene investigation towards the differentiation of melanoma and non-melanoma	Rapid and easy to perform	Test results need large genetic profile for differentiation process	Se: 69%, Sp: 75%	90
2	Cells from the stratum corneum is taken by means of tape stripping and detects the melanoma from nevi	Less resource consumption procedure	Produce itching effect after the procedure	Se: 100%, Sp: 88%	229
3	Different non-invasive techniques involved in detecting melanoma in which tape stripping act as excellent clinical information provider for melanoma detection using mRNA profiles	Easy procedure to perform	Investigation needs large set gene profile datasets	—	230
4	The best condition for tape stripping procedure was validated through the evaluation of the distribution of corneocytes, mass of stratum corneum (SC) removed and amount of protein removed using finger pressure, a 2 kg weight and a roller	Cost is less	Not used clinically due to conflicts in testing results	—	232
5	RNA from the stratum corneum is isolated to give 312 gene expressions for the detection of melanoma	Rapid and convenient method and patient friendly	Lack of standardized protocol and heterogeneous sampling	Se: 100%, Sp: 88%	233
6	Differentiation between lentigo maligna and lentigo maligna melanoma from solar lentigo, which is sometimes difficult with histology is easily performed by the extracted genetic information	Sensitive and specific for melanoma and can be able to detect genomic changes before morphological changes appear	Only few studies are available to prove the accuracy, sensitivity and specificity features of this technique	Se: 100%, Sp: 88%	234
7	RNA isolation, RNA amplification and array hybridization were performed to differentiate melanoma from dysplastic nevi	Accurate results are obtained at the preliminary stage of the tests	Need extra 15 to 20 melanoma sample for robust operation which causes local irritation to the patient	Acc: 100%	235
8	RNA extraction, synthesis of cDNA, probe labelling, array hybridization, quantitation of hybridization signals, reverse transcription polymerase chain reaction (RT-PCR), confirmation of markers were performed to differentiate the types of skin cancer	Earlier diagnosis of melanoma is performed	Can be performed only when the extracted particles are frozen deeply	—	236
9	One hundred and fifty pigmented lesions were tested using tape stripping toluidine blue (TSTB) method in the diagnosis of malignant melanoma and found the sensitivity and specificity	Early detection of malignant melanoma is performed in addition with ABCDE rule	This investigation takes time to collect data	Se: 68.7%, Sp: 74.5%	237
10	Preliminary investigation was designed to compare the gene expression profiles of melanoma and benign nevi	Epidermal genetic information retrieval (EGIR) has the potential to identify future biomarkers which would be useful in targeted therapy	Only few studies are available to prove the accuracy level	Se: 100%, Sp: 88%	238


### Multi photon scanning

**Fig. 18:**
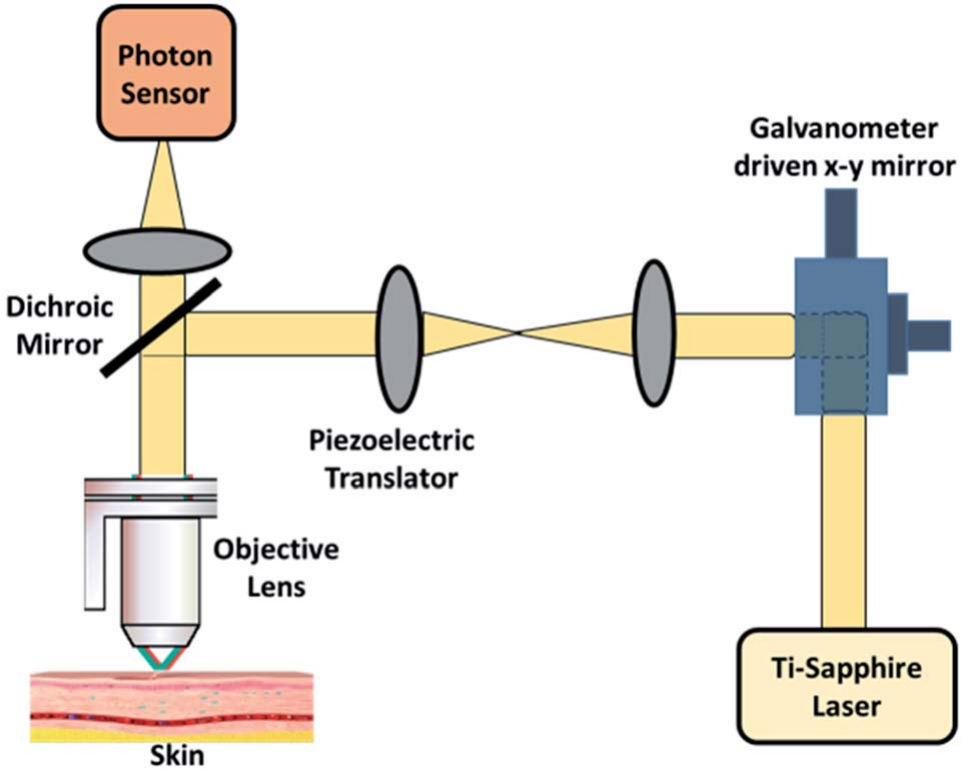
Multiphoton scanning technique. In the above image shown setup used two-photon excitation that allows imaging of living tissue up to one millimetre in depth.

Multiphoton scanning is also known as two-photon excitation microscopy, non-linear or two-photon laser scanning microscopy which is an alternative to confocal and deconvolution microscopy that provides distinct advantages for three-dimensional imaging.^239^ The principle of multiphoton scanning is the nonlinear interactions between photons and matter. Interaction of two photons with the same molecule at the same time occurs in two-photon excitation. This two-photon excitation causes fluorescence. Then, the single photon is required to process the fluorescence of right energy to interact with the molecule. Excitation is not produced when the two-photon excitation is used through the specimen, as the photon density is adequately high to cause two-photon absorption. Excitation occurs in the focal spot of the microscope.^240^ The complete setup is shown in Fig. 18. In particular, two-photon excitation excels at imaging of living cells, especially within intact tissues such as brain slices, embryos, whole organs, and even entire animals. Two-photon excitation microscopy provides great utility for dynamic imaging of living cells in thick specimens, such as intact tissue. The technique does many possible experiments in which conventional imaging cannot be performed.^241^

Researchers like Barry R. Masters, Peter T., C. Enrico Gratton, who worked on multiphoton laser scanning microscopy (MPLSM) demonstrated that the power entering the microscope can be controlled by Glan-Thompson polarizer which reduces power dissipation and increases the efficiency.^242^ In order to find the difference in accuracy for confocal laser scanning microscopy (CLSM) method and multiphoton microscopy, various histological tests were undertaken by Barry *et al.* and inferred that both methods have similar accuracy.^243^ However, resolution in terms of three-dimensional gene expressions and depths of tumor are more accurate in MPLSM.^244^ Later, Timothy P. Padera and colleagues found that epiluminescence property in MPLSM showed the better result in terms of gene expression.^245^ The major cause of skin cancer is UV rays which makes the skin to lose the collagen and elastin material in the skin are widely affected.^246^ Morphological altering due to skin aging is investigated by pathological characterization.^247^ Morphological features in 14 people who are non-melanoma are performed with MPLSM.^248^ Later 83 person were tested considering the morphological features of non-melanoma and melanoma skin cancer subjects, which showed a major difference and useful in diagnosis.^249^ Further to prove the accuracy of both CLSM and MPLCM in differentiating skin layer tumour, Martin Johannes Koehler and his colleagues performed pathological tests.^250^ MPLSM provides high-resolution fluorescence imaging, allowing visualization of cellular and subcellular structures of the epidermis and upper dermis.^251,252^ MPLSM based morphologic features of skin cancer images are comparable to traditional histopathology.^252^ Latest updates on multiphoton scanning for skin cancer diagnosis are summed in Table 13.

**Table 13:** Recent reports on multiphoton laser scanning microscopy techniques for skin cancer diagnosis

S. no	Signal used	Description	Merits	Demerits	Acc	Ref.
1	730 nm and 960 nm	Multiphoton excitation microscopy was used to image *in vivo* human skin auto fluorescence from the surface to a depth of −200 μm	Deepest layer of the tissue is observed and displayed in a 3D image	Photo damage occurs	—	242
2	80 MHz at 780 nm	Compared the tandem scanning reflected light confocal microscopy and multi-photon excitation microscopy for the observation of human skin *in vivo*. Both techniques gave similar results from the skin surface to the epidermal–dermal junction	Excitation can only occur in the focal plane, so out-of-focus bleaching is avoided	Minimal photo damage occurs	—	243
3	810–850 nm	Presented that the multiphoton laser-scanning microscope can provide high three dimensional resolution of gene expression and function in deeper regions of tumors	Lower photo toxicity due to the longer wavelengths	Cell damage occur due to three-photon excitation	—	244
4	780 nm	Intravital epifluorescence microscopy has provided powerful insight into gene expression, tissue pH, tissue pO2, angiogenesis, blood vessel permeability, leukocyte–endothelial interaction, molecular diffusion, convection and binding	1. Depth of imaging is increased	Cost is high	—	245
			2. Projection of three-dimensional structures onto a two-dimensional plane			
			3. Photo toxicity is reduced			
5	820 nm	Major features of aged skin include dryness, flaccidness, wrinkling, multiple, mostly benign neoplasms, signs of chronic UV damage, carcinogenesis, and functional deficiency. Based on collagen and elastin content in the skin, skin aging is defined	Second harmonic finding correlated with the histological findings in an *ex vivo* manner	Movement artefacts occur which affects the resulting image	—	246
6	750 nm and 850 nm	Pathological and physiological characterization conditions by MPLSM *in vivo* are performed to investigate and identify skin morphological alterations related to ageing	Useful for diagnostics of pathological conditions and their differentiation from ageing effects	Consumes time for cellular differentiation	—	247
7	Femtosecond pulsed laser at 780 nm	Describes about the morphologic features of human non-melanoma skin cancer obtained using MPLSM on freshly excised specimens from 14 patients. Emission from the skin was detected in the range of 450–530 nm, corresponding to the auto fluorescence of nicotinamide adenine dinucleotide, NADPH, keratin, elastin, collagen, and melanin, allowing us to visualize the cellular structures within the epidermis and upper dermis of the specimens	Deeper biological tissue can be viewed	Cost is high	—	248
8	Mode lock at 80 MHz, and tuning range at 750–850 nm	Laser radiation in the near infrared spectrum was used to image endogenous fluorophores by multiphoton excitation. Eighty-three melanocytic skin lesions have been investigated. The results showed distinct morphological differences in melanoma compared with melanocytic nevi	High accuracy	Cost is high	Acc: 97%	249
9	Tunable range at 750–850 nm and excited at 760 nm	Comparison of confocal microscopy and multiphoton tomography were performed. Both methods, CLSM and MPT, were found to be suitable for *in vivo* imaging of superficial skin layers	Both methods have high resolution and near-real time acquisition speed of the image	It was not possible to use same optical medium for all the methods	—	250
10	532 nm	Multimodal imaging, the combination of two-photon excited fluorescence (TPEF), and second harmonic generation (SHG), which have high diagnostic potential. Multimodal imaging generates molecular contrast, but to use this technique in clinical practice, the optical signals must be translated into diagnostic relevant information	Increased reliability	Cost is high	—	251
11	Femtosecond pulsed laser at 780 nm	Demonstrated MPLSM as a diagnostic tool for superficial skin cancers, such as BCC, SCC *in situ*, and skin. The morphologic features observed in MPLSM images of skin tumors are comparable to traditional histopathology	Imaging depth is increased here	Loss of signal occur during focusing	—	252

## Conclusion

This article briefly summarizes the techniques which deal with the non-invasive approach towards the detection of skin cancer. The different techniques that use various modalities to diagnose the skin cancer are demonstrated with recent updates. However, every system equally shares the advantages and disadvantages in all aspects. We found that the use of TBP surveillance is must in skin lesions and dysplastic nevi which show the result but these results vary according to the experience of the doctor that lead to misinterpretation in some cases while differentiating BCC and melanoma. Also, the age of onset and frequency at which it should be performed remains unclear. This makes limited data to diagnose. Some studies show that there was no evidence in practicing photography as a screening tool in the United States by the dermatologists; instead, they prefer a dermoscopy. A handheld dermoscope is implemented in dermoscopy that provides at least 10-fold magnification of the skin lesions, and almost all primary cares adopt this mode of diagnosing. And no study reported that the survival of melanoma has increased due to dermoscopy evolution. This is mainly due to the same fact that the result varies according to the experience of physician that makes less trust in public towards the clinical screening. As far as the reviews from several abstracts, this could be changed by using a combination of both photography and dermoscopy in heterogeneous aspects that resolves all cons of the conventional techniques and provide an accurate result. The efficiency of the results is based on the usage of algorithms, devices, training and practitioner's experience.

Nowadays to avoid false positive results dermatologist use confocal laser scanning microscopy which aid in the assessment of skin lesions by providing high resolution, high sensitivity, high specificity images that are similar to the histopathological images. The only downside of this technique is the high cost that could not be undertaken or affordable by economically backward people. Unlike other techniques, this is unique which is not engaged in comparison with other techniques (dermoscopy) trials to prove the accuracy of results. However, the reported outcomes were mainly focused on the lesion characterizations of CSLM and its test accuracy. Also, imaging modalities like high-frequency sonography, confocal microscopy and OCT have limited availability and are usually found in research units and selected dermatology departments. They are predominantly practiced on an experimental and research basis. Despite their limitations, these high-resolution techniques can give us valuable information on superficial BCC. Nevertheless, awareness of their penetration intricacies must be kept in mind because they may leave cancerous lesions that affect the lower dermis and deeper layers out of their fields of view. Many other modalities like high-frequency ultrasound sonography, PDD, Raman spectroscopy, fluorescence spectroscopy and THz spectroscopy are still in limited practice in case of skin cancer detection. Techniques like thermography which needs optimum instrumentation for the recording purpose and widely affected by the external noise though it provides a thermogram at high resolution. In case of MPLSM, bio-electrical impedance, OCT, tape stripping, multispectral imaging and fully automated computer-based analysis are considered as investigational that need clinical approval in different usage modalities. Also there are not much evidences or trials undertaken to prove the accuracy with conventional histological determination.

### Future directions

The delay of implementing dermoscopy widely in the hospital and other healthcare is due to the lack of training among the physicians. This made an adverse impact in getting such product to the market field. However, usage of higher end resolution camera and instrumentation set up must provide a better result that is indirectly failed to achieve due to insufficient training. Thus proper training, knowledge and experience sharing of practitioners are must for better diagnosis. The use of computer-aided diagnosis in finding the skin lesions could be furtherly enhanced using various pre-processing techniques and segmentation method that would be more informative and satisfying the needs. It is the need for analyzing various attributes and can also be included in dermoscopic techniques so that it is easier to link with computer-aided analysis. The optical methods like OCT and PDD are still under investigation stages; this could be improved by defining the borders of suspicious lesions particularly in non-melanoma skin cancer and the common skin disorders. Further exploration is required in using the laser for detecting melanoma.

The features of the future systems should be (i) low cost, (ii) compact and portable, (iii) less diagnosis time, (iv) comfortable (no biopsy), (v) high sensitivity and accuracy. Combination of multiple techniques should be explored for high-quality output and accuracy. Clinicians, researchers and practitioners should step forward towards arriving at a standard protocol and knowledge sharing database, thus help in providing better healthcare relating to skin cancer.

## Conflicts of interest

There are no conflicts to declare.

## Abbreviations

Acc: Accuracy
BCC: Basal cell skin cancer
CNN: Convolutional neural network
MSOT: Multi-spectral optoacoustic tomography
MPT: Multi photon tomography
OCT: Optical coherence tomography
ODT: Optical doppler tomography
PDT: Photodynamic therapy
PAI: Photoacoustic imaging
PDD: Photodynamic diagnosis
RCM: Reflectance confocal microscopy
Res: Resolution
Se: Sensitivity
Sp: Specificity
SCC: Squamous cell carcinoma
THz: Tetrahertz
TBP: Total body photography
TPI: Tetrahertz pulsed imaging

## Supplementary Material
